# Silencing of METTL3 effectively hinders invasion and metastasis of prostate cancer cells

**DOI:** 10.7150/thno.61178

**Published:** 2021-06-11

**Authors:** Yabing Chen, Chun Pan, Xiaotong Wang, Dihui Xu, Yuhan Ma, Jianhang Hu, Peilin Chen, Zou Xiang, Qiu Rao, Xiaodong Han

**Affiliations:** 1 Immunology and Reproduction Biology Laboratory & State Key Laboratory of Analytical Chemistry for Life Science, Medical School, Nanjing University, Nanjing 210093, China.; 2 Jiangsu Key Laboratory of Molecular Medicine, Nanjing University, Nanjing 210093, China.; 3 Department of Pathology, Jinling Hospital, Nanjing University School of Medicine, Nanjing 210002, China.; 4 Department of Health Technology and Informatics, Faculty of Health and Social Sciences, The Hong Kong Polytechnic University, Hung Hom, Kowloon, Hong Kong China.

**Keywords:** METTL3, m^6^A, prostate cancer, metastasis, therapeutic strategies

## Abstract

**Background**: Since primary prostate cancer (PCa) can advance to the life-threatening metastatic PCa, exploring the molecular mechanisms underlying PCa metastasis is crucial for developing the novel targeted preventive strategies for decreasing the mortality of PCa. RNA N^6^-methyladenosine (m^6^A) is an emerging regulatory mechanism for gene expression and its specific roles in PCa progression remains elusive.

**Methods:** Western blotting, quantitative real-time PCR and immunohistochemical analyses were used to detect target gene expression in PCa cells *in vitro* and prostate tissues from patients. RNA immunoprecipitation was conducted to analyze the specific binding of mRNA to the target protein. Migration and invasion assays were used to assess the migratory capacities of cancer cells. The correlation between target gene expression and survival rate of PCa patients was analyzed based the TCGA database.

**Results:** We found that total RNA N^6^-methyladenosine (m^6^A) modification levels were markedly upregulated in human PCa tissues due to increased expression of methyltransferase like 3 (METTL3). Further studies revealed that the migratory and invasive capacities of PCa cells were markedly suppressed upon METTL3 knockdown. Mechanistically, METTL3 mediates m^6^A modification of USP4 mRNA at A2696, and m^6^A reader protein YTHDF2 binds to and induces degradation of *USP4* mRNA by recruiting RNA-binding protein HNRNPD to the mRNA. Decrease of USP4 fails to remove the ubiquitin group from ELAVL1 protein, resulting in a reduction of ELAVL1 protein. Lastly, downregulation of ELAVL1 in turn increases ARHGDIA expression, promoting migration and invasion of PCa cells.

**Conclusions:** Our findings highlight the role of METTL3 in modulating invasion and metastasis of PCa cells, providing insight into promising therapeutic strategies for hindering PCa progressing to deadly metastases.

## Introduction

Prostate cancer (PCa) is the most frequently diagnosed cancer and the second leading cause of cancer-related death among males 1, 2. Given the importance of androgen receptor in prostate carcinogenesis, those patients with low-grade localized tumors are sensitive to androgen-deprivation therapies and are generally curable by surgery, chemotherapy and radiotherapy 3. However, once primary PCa advances to a deadly metastatic PCa and acquire resistance to androgen-deprivation therapies, the 5-year survival for patients drop to 30% 4, 5. Thus, hindering PCa metastasis can improve the prognoses of patients. Considering cancer metastasis is a highly complex process facilitated by a series of key events, and thus understanding the underlying molecular mechanism associated with these events will be beneficial to clinical management 2, 6, 7. Moreover, identifying and targeting those vital genes involved in PCa metastasis will be essential for developing future therapeutic strategies to control PCa metastatic spread.

N^6^-methyladenosine (m^6^A), the most abundant internal modification in eukaryotic mRNA, mediates gene expression by regulating RNA stability, subcellular localization, translation efficiency, and alternative splicing 8, 9. m^6^A modification is usually embedded within the consensus motif DRACH (D = A, G, U; R = A, G; H= A, C, U) and usually happens around stop codons 9. Moreover, m^6^A modification is a dynamic and reversible process, during which the methyltransferase complex consisting of methyltransferase-like 3 (METTL3) and METTL14 catalyze m^6^A modification; however, alkB homolog 5 (ALKBH5) and fat mass and obesity-associated protein (FTO) reverse the m^6^A modifications 9. Moreover, the prerequisite for m^6^A exerting its effects is recruiting m^6^A-binding proteins to m^6^A sites, among of which YTHDF2 binds to and induce degradation of m^6^A-modified mRNAs by recruiting some RNA-binding proteins to the mRNAs 10, 11.

Recently, growing evidences have supported that m^6^A modification regulates tumor progression including gastric cancer 12, endometrial cancer 13, liver cancer 14, and lung cancer 15. Thus, we wonder whether m^6^A also plays a key role in modulating PCa progression. In the present study, we observe that METTL3 is upregulated in human androgen-independent PC3 and DU145 cells as well as androgen-sensitive LNCaP cells, accompanied by increased cellular m^6^A levels. We also find that testosterone effectively induces METTL3 expression and m^6^A modification in LNCaP cells but not PC3 and DU145 cells. Further studies demonstrate that m^6^A facilitates PCa metastasis by modulating ARHGDIA expression via the METTL3-USP4-ELAVL1 cascade.

## Materials and Methods

### Main reagents and cell culture

RPMI 1640 medium, fetal bovine serum (FBS), and penicillin-streptomycin were purchased from Gibco (Grand Island, NY). The protein synthesis inhibitor cycloheximide (HY-12320), proteasome inhibitor MG132 (HY-13259), DNA transcription inhibitor actinomycin D (HY-17559), and Bobcat339 (HY-111558A) were obtained from Medchemexpress (Monmouth Junction, NJ). The detailed information about antibodies used in this study was shown in **Table S1**. PC3, DU145, and LNCaP cells were obtained from ATCC, and were cultured in RPMI 1640 medium supplemented with 10% FBS and 1% penicillin-streptomycin.

### Quantitative real-time PCR (qRT-PCR) and RNA stability analyses

RNA was isolated using Trizol reagent (15596018) (Invitrogen, Carlsbad, CA) following the manufacturer's instructions, and cDNA was generated with iScript cDNA Synthesis Kit (170-8890) (BioRad, Hercules, CA). The qRT-PCR assay was performed using ChamQ SYBR Master Mix (Q311-02) (Vazyme, Nanjing, China) on ViiA 7 Q-PCR System (Applied Biosystems, Waltham, MA). All primers used in the present study were listed in **Table S2**, and *GAPDH* was used as an internal control to measure the relative mRNA levels of targeted genes. RNA stability assay were performed as described previously 16.

### Western blotting and coimmunoprecipitation (Co-IP) analyses

Total protein lysates were isolated with RIPA buffer (P0013C) (Beyotime, Shanghai, China), and the concentration of protein was determined with BCA Protein Quantification Kit (Vazyme, E112). Western blotting was performed as described previously 16, and the intensity of the western blotting bands was quantified using Image J software. Furthermore, GAPDH was chosen as marker protein in this study. For protein stability assay, cells were treated with cycloheximide at 100 μg/mL for indicated times, after which protein levels were determined by western blotting. Moreover, Co-IP was performed as described previously 14.

### m^6^A RIP-qRT-PCR analyses and measurement of cellular m^6^A levels

To assess the m^6^A modification levels of USP4 mRNA, m^6^A RIP was performed using Magna MeRIP^TM^ m^6^A kit (17-10499) (Millipore Sigma, Billerica, MA) according to manufacturer's instructions with a slight modification. Briefly, the isolated RNAs were fragmented with RNA fragmentation buffer. After saving one tenth of the total RNA as input, the remaining RNAs were immunoprecipitated with m^6^A antibody-conjugated magnetic beads. m^6^A-modified RNAs were washed with immunoprecipitation buffer for three times and then eluted with elution buffer. Total RNAs from elution buffer were recovered with Trizol reagent and then subjected to qRT-PCR assays. The specific primer information about USP4 was listed in **Table S3**. The relative m^6^A modification levels of *USP4* at different m^6^A modification sites were normalized to input. Moreover, EpiQuik m^6^A RNA Methylation Quantification Kit (P-9005-96) (Epigentek, Farmingdale, NY) was chosen to measure total cellular m^6^A modification levels according to manufacturer's instructions.

### RNA-immunoprecipitation (RIP)-qRT-PCR analyses

RIP analyses were performed as previously established protocols 16. Briefly, cells were firstly lysed with RIP lysis buffer containing protease and RNase inhibitor, after which cell lysate supernatant was incubated with magnetic beads coated with antibodies against rabbit immunoglobulin G, YTHDF2, HNRNPD, or ELAVL1 overnight at 4 °C. The beads were then washed with IP buffer for three times, followed by being treated with proteinase K (Millipore Sigma, 107393) at 65 °C for 0.5 h. Total RNA from the supernatant was recovered with Trizol reagent. The association between *USP4* transcript and target proteins were measured by qRT-PCR assay, and the data were normalized to input. Specific primer information was listed in **Table S4**.

### Human PCa tissue specimens

In this study, a total of 25 pairs of PCa tissues and adjacent normal tissues were collected from department of pathology at Jinling hospital (Nanjing, China) with appropriate informed consent from patients. Clinical information about these patients was provided in **Table S5**.

### Immunohistochemical analyses

The human and mouse sections need to be dewaxed and rehydrated, followed by antigen retrieval using 10 mM citrate buffer. After being treated with 3% H_2_O_2_, the sections were immersed with primary antibodies overnight at 4 °C and then incubated with HRP-conjugated secondary antibody for 1 h at room temperature. The immune complexes were examined using the diaminobenzidine (G1212-200T) (Servicebio, Wuhan, China) according to manufacturer's instructions. The Image-Pro Plus software (Media Cybernetics, MD) was chosen to quantify the protein levels by calculating the integrated optical density per stained area (IOD/area).

### Migration and invasion assays

PC3, DU145, and LNCaP cells were plated onto 24-well transwell chambers with polycarbonate membranes (353097) (Corning Life Science, Acton, MA) to assess cell migration and invasion capacities with or without Matrigel (Corning Life Science, 354234). The outer chamber was filled with the complete medium and the upper chamber was seeded with cells at a density of 5 × 10^5^ cells/well in serum-free medium. After being cultured for 12 h, cells adhering to the underside of the chamber were fixed with 4% paraformaldehyde for 5 min, and then incubated with 100% methanol for 20 min. Finally, migrating cells on the lower surface of the membrane were stained with crystal violet for 15 min, and the cells were counted under an optical microscope.

### Plasmid construction and transfection

Lentiviral vectors expressing *METTL3*-specific, *ELAVL1*-specific, or *USP4*-specific shRNA were constructed by OBiO (Shanghai, China). The target sequences of shRNA are as following: sh-*METTL3*#1: 5'-CTGCAAGTATGTTCACTATGA-3'; sh-METTL3#2: 5'-CGTCAGTATCTTGGGCAAGTT-3'; sh-*ELAVL1*: 5'-GAGAACGAATTTGATCGTCAACT-3'; sh-*USP4*: 5'-GCCCAGAATGTGCTAAGGTTTCT-3'; sh-control: TTCTCCGAACGTGTCACGT. The METTL3 expression plasmid was purchased from GENECHEM (Shanghai, China). HA-Ubiquitin plasmid was obtained from Addgene (18712) (Watertown, MA). The full-length human *ARHGDIA* cDNA was cloned into the pcDNA3.1 vector (VT1001) (YouBio, Changsha, China) to generate expression plasmid. The USP4 expression plasmid was generated by cloning the full-length *USP4* cDNA into pcDNA3.1 vector with HA tag (YouBio, VT8001). Wild-type or mutated *USP4*-CDS sequence (2600 ~ 2900) was cloned into pmirGLO luciferase vector (YouBio, VT1439). For sg*METTL3* expressing cells, the target sequences (5'-AGAGTCCAGCTGCTTCTTGT-3') were cloned into pLentiCRISPR v2 17. PCa cells were transfected with indicated plasmids. After 48 h of transfection, PC3, DU145, and LNCaP cells were selected with puromycin at 6 μg/ml, 2 μg/ml, and 2 μg/ml, respectively. For the METTL3 rescue experiment, the METTL3 expression plasmid was transfected into those PCa cells with METTL3 knockout.

### Methylation-specific PCR

DNA from RWPE-1 cells, PC3 cells, DU145 cells, and LNCaP cells were extracted using DNA extraction kit (DP304) (TIANGEN, Beijing, China). The sodium bisulfite DNA treatments were conducted using a DNA bisulfite conversion kit (TIANGEN, DP215) according to the manufacturer's instructions. PCR amplifications were performed using Phanta® UC Super-Fidelity DNA Polymerase (Vazyme, P507-01) according to the manufacturer's instructions. The PCR primers for methylation-specific PCR were listed in **Table S6**.

### Luciferase reporter assay

Cells seeded in 12-well plates were transfected with the pmirGLO luciferase reporter vector fused with or without the wild-type or mutated USP4-CDS. The firefly luciferase and Renilla luciferase activity in each well were measured by a dual-luciferase reporter assay system (E1910) (Promega, Madison, WI) and the relative luciferase activity was further normalized to Renilla luciferase activity.

### Animal experiments

Male BALB/c nude mice and male SCID mice aged 4-5 weeks were purchased from the Model Animal Research Center of Nanjing University, Nanjing, China and were maintained in a pathogen-free animal facility at least 1 week before use. A total of 1 × 10^6^ PC3 cells or DU145 cells suspended in a mixture of 100 μL PBS and Matrigel were subcutaneously injected into BALB/c nude mice. Tumor weight were measured 2 months after the engraftment. To evaluate the role of METTL3 in tumor metastasis, PC3 cells with or without knockdown of METTL3 were injected into SCID mice through the tail vein (1 × 10^6^ cells per mouse). After eight weeks, mice were sacrificed and their lung tissues were collected for subsequent analyses. The whole experiments on mice were approved by the Animal Care and Use Committee of Nanjing University under the animal protocol number SYXK (Su) 2009-0017.

### Flow cytometric analyses

Cell cycle distribution was determined by flow cytometric analyses to investigate whether METTL3 knockdown affect the proliferation of PCa cells. Briefly, PC3 cells were incubated with 500 μl PI (550825) (BD Pharmingen, San Jose, CA) for 30 min, after which cellular PI absorbance was measured by a FACSCalibur flow cytometer (BD Biosciences, San Jose, CA).

### Statistical analyses

The data were analyzed for statistical significance with SPSS 18.0 (SPSS, Chicago, IL). All statistical tests were two-sided, and* p* < 0.05 was considered statistically significant. Differences between groups were tested by One-way analysis of variance (ANOVA) followed by Duncan's *post hoc* test. Data violated the assumption of homogeneity of variances, an alternative Welch's ANOVA with Dunnett's T3 test was carried out.

## Results

### PCa is associated with upregulated METTL3 expression

**Figure 1:**
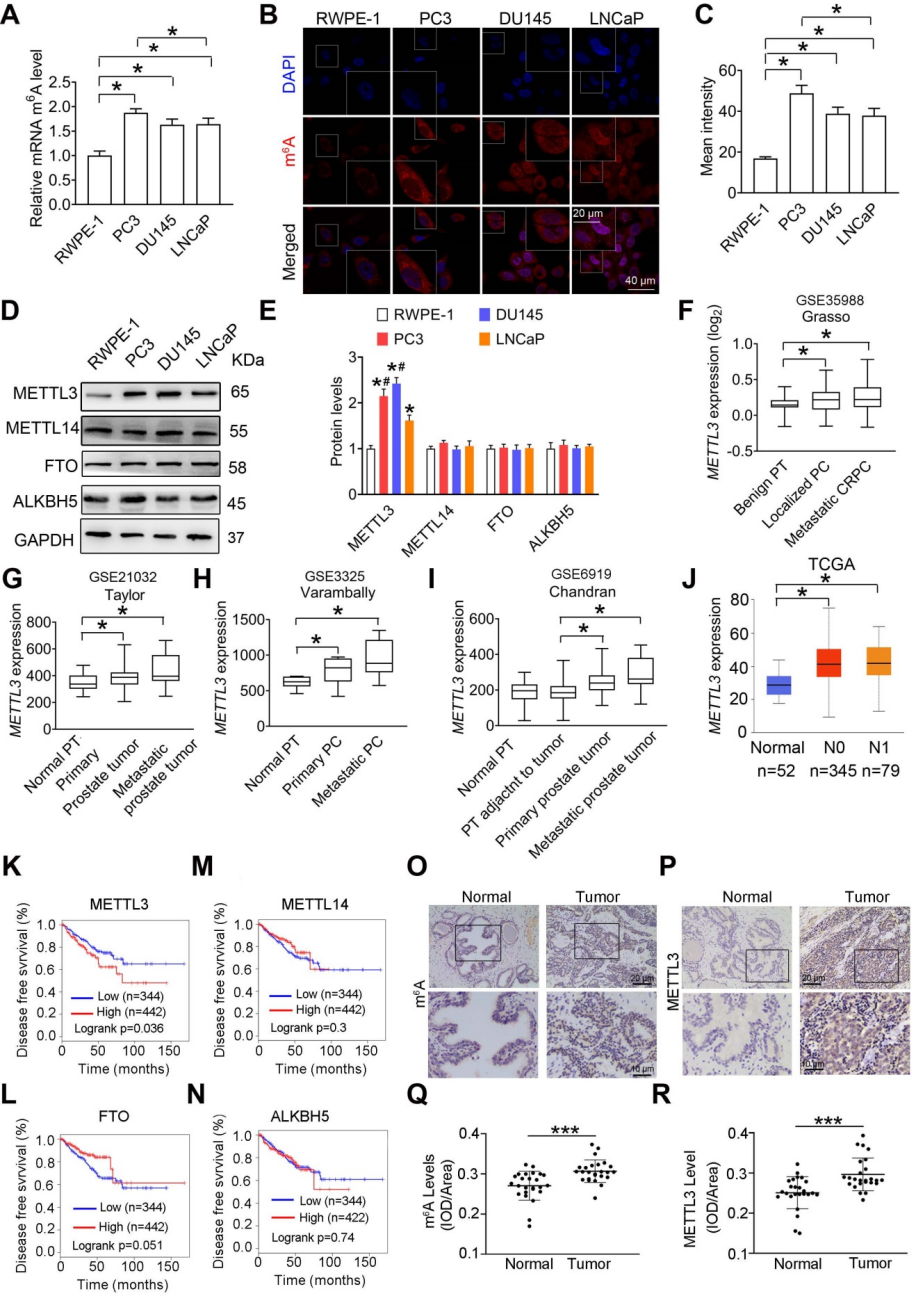
** The m^6^A mRNA methylation mediates prostate cancer (PCa) progression. A,** Relative m^6^A levels in RWPE-1, PC3, DU145, and LNCaP cells were measured by ELISA-based m^6^A quantitative analyses. Data were presented as means ± SEM (n = 3), *p < 0.05. **B**-**C,** Cellular m^6^A levels were assessed by immunofluorescence staining, and fluorescence intensity was presented as means ± SEM (n = 3), *p < 0.05. **D**-**E,** Protein levels of m^6^A regulatory enzymes in indicated cells were determined by western blotting and quantitatively analyzed. Data were presented as means ± SEM (n = 3), * *p* < 0.05 vs. the RWPE-1 cells. **F**-**J,** The *METTL3* mRNA levels were analyzed based on 4 different GSE datasets and the TCGA database (*p < 0.05). PT: prostate tissue; PC: prostate cancer; CRPC: castration-resistant prostate cancer. **K**-**N,** Association of METTL3, FTO, METTL14, and ALKBH5 mRNA levels with survival of PCa patients was performed using Kaplan-Meier survival curve analysis methods based on the TCGA database. **O**-**R,** Immumohistochemical staining was performed to evaluate the m^6^A levels and expression of METTL3 in 25 paired human PCa tissues and their adjacent normal prostate tissues. The m^6^A levels (**Q**) and METTL3 protein levels (**R**) were analyzed by calculating the integrated optical density per area (IOD/area). Data were presented as means ± SEM (n = 25), *** *p* < 0.001.

In this study, we measured an increase of m^6^A levels in human androgen-independent PC3 and DU145 PCa cells as well as androgen-sensitive LNCaP cells in comparison to RWPE-1 cells, a normal adult epithelial prostate cell line **(Figure 1A**-**C)**. We next examined upregulated expression of METTL3 in PCa cells, and no significant changes about METTL14, FTO, and ALKBH5 were observed **(Figure 1D**-**E)**. Moreover, in three PCa cell lines, relatively lower METTL3 and m^6^A levels were examined in androgen-sensitive LNCaP cells **(Figure 1D**-**E)**. It is the nuclear but not cytoplasmic-localized METTL3 mediates m^6^A deposition on nuclear RNA 9. As predicted, we examined marked enrichment of METTL3 but not METTL14, FTO, and ALKBH5 in the nuclei of PCa cells relative to RWPE-1 cells **(Figure S1)**.

Additionally, we found that *METTL3* mRNA levels were increased in primary prostate tumor and metastatic tumor tissues in comparison to normal prostate tissues** (Figure 1F**-**I)** by analyzing several publicly available Genomic Spatial Event (GSE) databases 18-20. Through analyzing The Cancer Genome Atlas (TCGA) dataset (http://ualcan.path.uab.edu/ index.html) 21, we further confirmed that *METTL3* mRNA levels were significantly increased in PCa tissues **(Figure 1J)**. Survival curve analyses (gepia.cancer-pku.cn) revealed patients with higher *METTL3* mRNA levels exhibited much shorter relapse-free survival rate** (Figure 1K)** 22; however, no significant correlation between METTL14, FTO, or ALKBH5 expression and survival rate was observed** (Figure 1L**-**N)**.

Moreover, we observed upregulated m^6^A levels in human PCa tissues relative to normal tissues, accompanied by a significant increase of METTL3 protein levels **(Figure 1O**-**R)**; however, no remarkable changes about METTL14, ALKBH5, and FTO were observed **(Figure S2)**.

### METTL3 is required for invasion and migration of PCa cells

**Figure 2:**
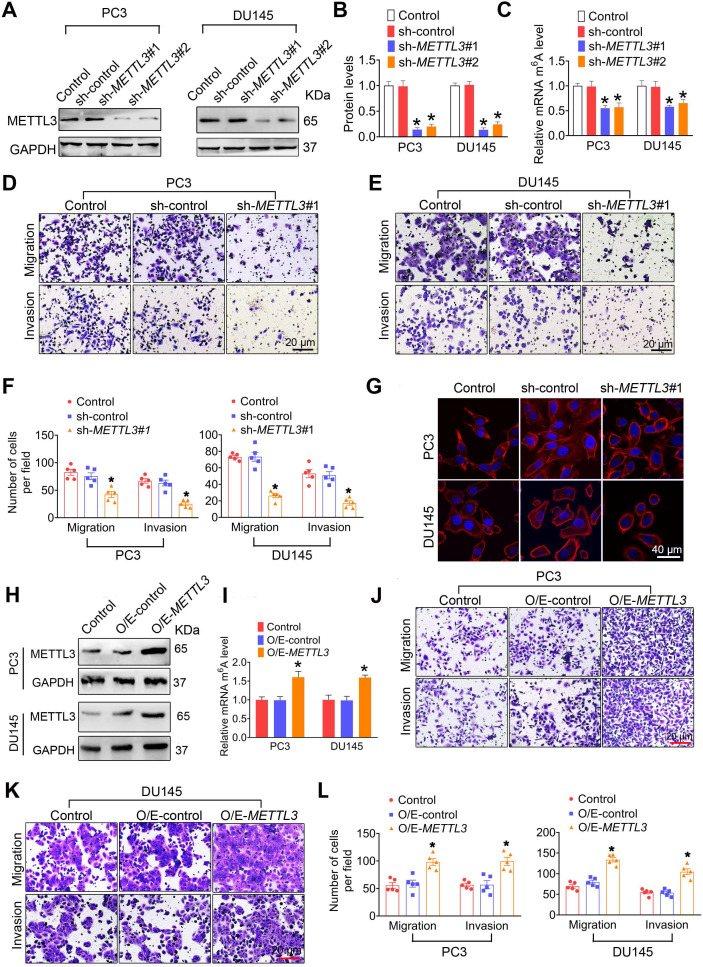
** METTL3 promotes prostate cancer (PCa) metastasis. A-C,** Two independent shRNA sequences targeting *METTL3* (sh-*METTL3*#1 and sh-*METTL3*#2) were separately transfected into PC3 or DU145 cells. Protein levels of METTL3 were determined by western blotting and quantitatively analyzed (**A, B**), and cellular m^6^A levels were measured by ELISA-based m^6^A quantitative analyses (**C**). **D-F,** The migration and invasion abilities of indicated cells were evaluated. Representative images (**D, E**) and quantification (**F**) of the cell migration and invasion assay results were shown. **G** PCa cells were stained with rhodamine phalloidin (red) and DAPI (blue). **H-L,** Overexpression constructs of *METTL3* (O/E-*METTL3*) were stably transfected into PC3 and DU145 cells, respectively. Protein levels of METTL3 were determined by western blotting (**H**), and cellular m^6^A levels were measured by ELISA-based m^6^A quantitative analyses (**I**). The migration and invasion abilities of indicated cells were assessed, and the representative images of the cell migration and invasion assay results were shown (**J-L**). Data were presented as means ± SEM where relevant, *p < 0.05 vs. the control cells.

In this study, we established stable METTL3-knockdown PCa cells with two independent shRNA sequences (sh-*METTL3*#1 and sh-*METTL3*#2)** (Figure 2A**-**B and Figure S3A**-**B)**. As predicted, knockdown of METTL3 significantly reduced m^6^A modification levels in PCa cells **(Figure 2C and Figure S3C)**. Considering that sh-*METTL3*#1 exhibited higher efficiency than sh-*METTL3*#2 in reducing METTL3 protein levels, and thus we chosen sh-METTL3#1 for further analyses. Moreover, we observed that METTL3 knockdown did not affect FTO, ALKBH5, and METTL4 levels in PCa cells **(Figure S3D**-**E)**.

We next found that METTL3 knockdown markedly inhibited migration and invasion of PC3 [42.2 ±10.89 vs. 82.6 ± 10.69 for migration assay, 25.2 ± 6.05 vs. 67.0 ± 7.97 for invasion assay], DU145 [26.20 ± 4.50 vs. 73.2 ± 3.54 for migration assay, 17.4 ± 5.31 vs. 53.0 ± 9.76 for invasion assay], and LNCaP cells [15.6 ± 2.96 vs. 33.6 ± 6.83 for migration assay, 12.2 ± 3.49 vs. 35.8 ± 5.71 for invasion assay] **(Figure 2D**-**F and Figure S3F**-**G)**. Consistedly, phalloidin-staining assay revealled that lamellipodia were formed depending on METTL3 expression in PCa cells** (Figure 2G)**. Moreover, we observed that METTL3 overexpression brought about increased m^6^A modification levels **(Figure 2H**-**I and Figure S3H**-**J)**, and concurrent enhanced migratory and invasive capacities in PC3 [98.2 ± 12.12 vs. 55.6 ± 10.23 for migration assay, 99.2 ± 14.52 vs. 56.6 ± 6.28 for invasion assay], DU145 [133.6 ± 10.89 vs. 69.4 ± 8.71 for migration assay, 104.6 ± 14.22 vs. 54.6 ± 6.97 for invasion assay], and LNCaP cells [44.2 ± 5.08 vs. 31.2 ± 6.24 for migration assay, 38.6 ± 4.54 vs. 28.8 ± 6.43 for invasion assay] **(Figure 2J**-**L and Figure S3K**-**L)**. To further confirm the role of METTL3 in mediating the PCa metastasis, we transfected the *METTL3* expression plasmid into those PCs cells with knockout of METTL3, and we found that *METTL3* overexpression effectively rescue the *METTL3* deficiency-induced suppressed migratory and invasive capacities of PCa cells** (Figure S4)**.

In this study, we found that testosterone robustly induced METTL3 expression and upregulation of cellular m^6^A levels in androgen-sensitive LNCaP cells but not androgen-independent PC3 and DU145 cells **(Figure S5A-C)**. Moreover, we identified that testosterone markedly upregulated migratory and invasive abilities of LNCaP but not PC3 and DU145 cells **(Figure S5D**-**H)**.

### ARHGDIA is an METTL3 downstream effector for PCa metastasis

**Figure 3:**
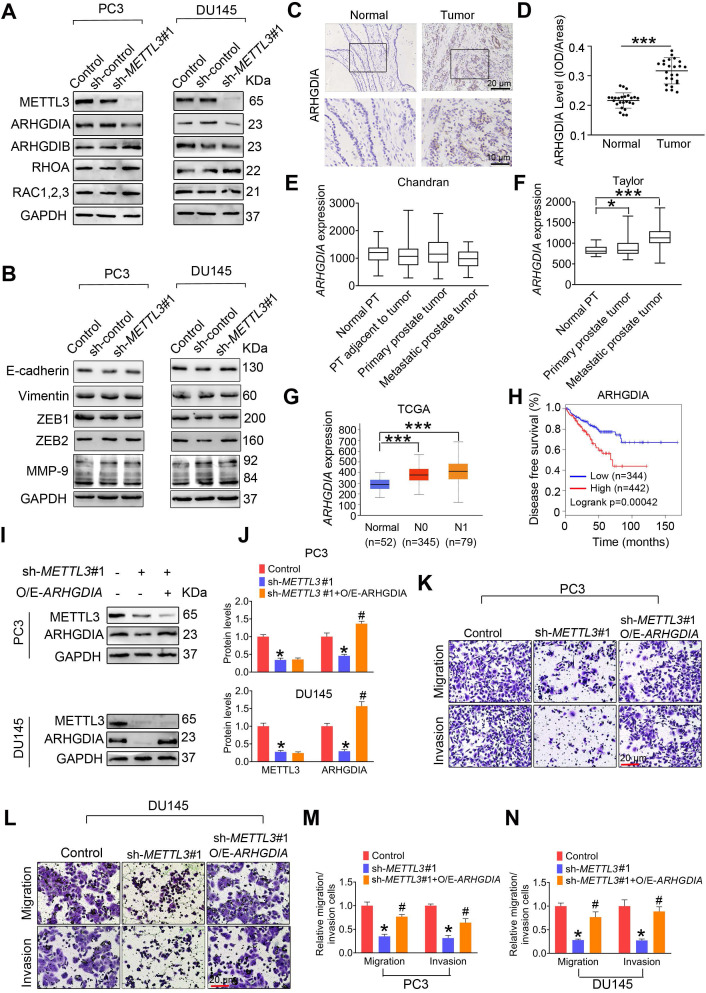
** ARHGDIA mediates METTL3 promotion of prostate cancer (PCa) metastasis. A**-**B,** PC3 and DU145 cells were transfected with shRNA targeting *METTL3* (sh-*METTL3*), respectively. Western blotting was used to examine protein expression of invasion- and EMT-related genes. **C-D,** Immumohistochemical staining was performed to evaluate the expression of ARHGDIA in 25 paired human PCa tissues and their adjacent normal prostate tissues, and ARHGDIA protein levels were analyzed by calculating the integrated optical density per area (IOD/area). Data were presented as means ± SEM (n = 25), *** *p* < 0.001. **E**-**G,** The *ARHGDIA* mRNA levels were analyzed in 2 different GSE datasets and the TCGA database, *p < 0.05. PT: prostate tissue. **H,** Correlation between *ARHGDIA* mRNA expression and survival of PCa patients was performed using Kaplan-Meier survival curve analysis methods based on the TCGA database. **I**-**N,** PCa cells were transfected with sh-*METTL3*#1 before transfection with pcDNA3.1-*ARHGDIA* (O/E-*ARHGDIA*). Protein levels of ARHGDIA were determined by western blotting and quantitatively analyzed (**I, J**). The migration and invasion abilities of indicated cells were evaluated. Representative images (**K, L**) and quantification (**M, N**) of the cell migration and invasion assay results were shown. Data were presented as means ± SEM (n = 3), * *p* < 0.05 vs. the control cells, # *p* < 0.05 vs. the sh-*METTL3*-treated cells.

We assessed the effects of METTL3 knockdown on the expression of invasion- and migration-related proteins, including Rho GDP dissociation inhibitor α (ARHGDIA), Rho GDP dissociation inhibitor β (ARHGDIB), ras-related C3 botulinum toxin substrate 1, 2, 3 (RAC1,2,3), matrix metalloproteinase-9 (MMP-9), and ras homolog gene family, member A (RHOA) 23-27. The results desmontrated that METTL3 knockdown markedly reduced expression of ARHGDIA **(Figure 3A)**. Moreover, METTL3 knockdown did not affect expression of epithelial-mesenchymal transcition (EMT)-related genes, including E-cadherin, vimentin, Zinc finger E-box binding homeobox 1 (ZEB1), and ZEB2 **(Figure 3B)**.

We found that ARHGDIA protein levels were increased in human PCa tissues relative to adjacent normal tissues **(Figure 3C**-**D)**. Additionally, *ARHGDIA* mRNA levels were also markedly upregulated in PCa tissues **(Figure 3E**-**G)**. Survival curve analyses revealed PCa patients with higher *ARHGDIA* expression exhibited much shorter relapse-free survival **(Figure 3H)**. To further verify the biological contribution of ARHGDIA in METTL3-mediating invasion of PCa cells, the constructs that express ARHGDIA were stably transfected into PCa cells **(Figure 3I**-**J)**. We observed that ectopic expression of ARHGDIA effectively reversed METTL3 knockdown-induced reduced migrative and invasive capacities in PC3 and DU145 cells **(Figure 3K**-**N)**.

### METTL3 stabilizes *ARHGDIA* mRNA by regulating ELAVL1 expression

**Figure 4:**
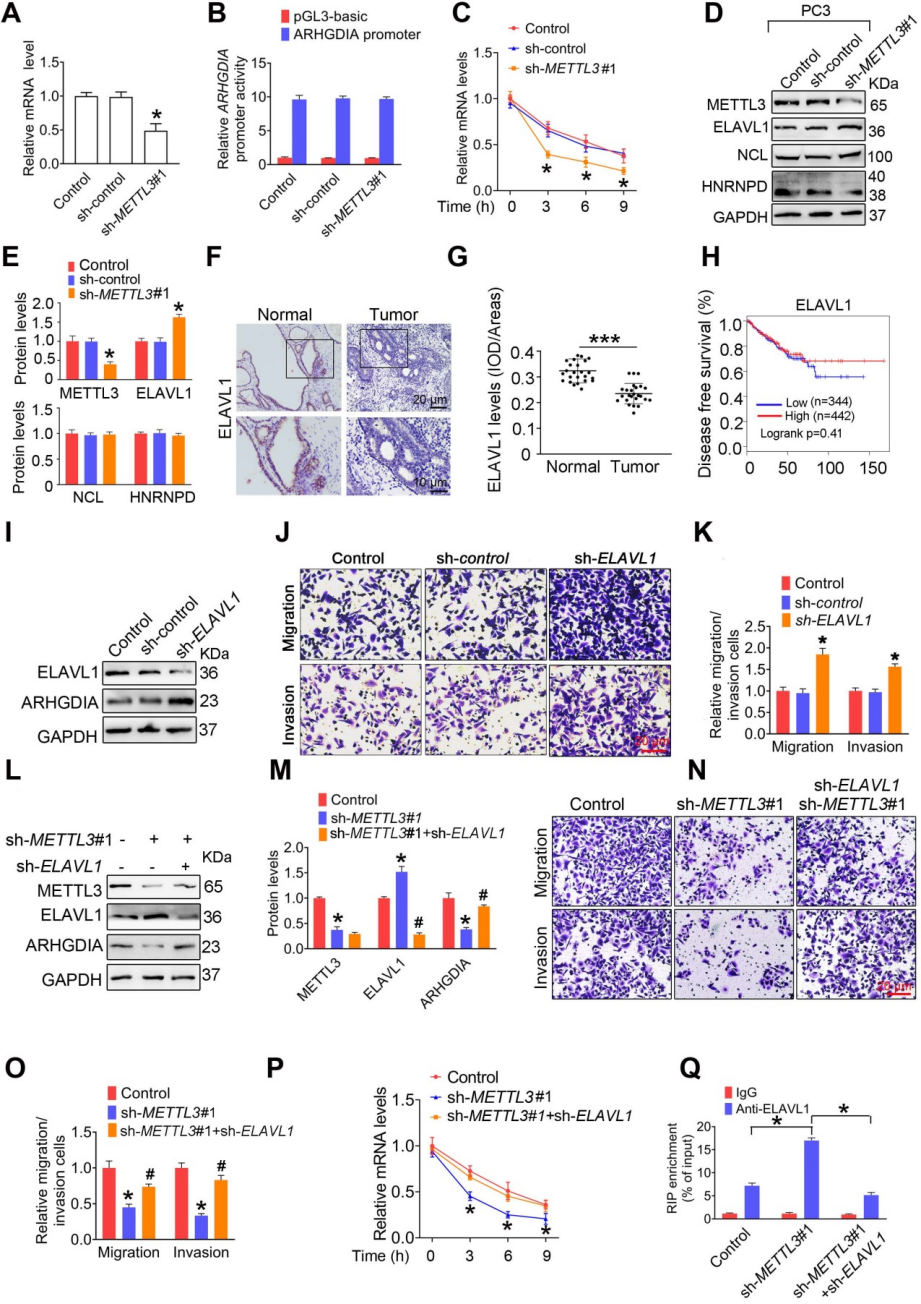
** ELAVL1 reduces *ARHGDIA* mRNA stability and prostate cancer (PCa) metastasis. A**-**E,** PC3 cells were transfected with shRNA targeting *METTL3* (sh-*METTL3*). The *ARHGDIA* mRNA levels in indicated cells were determined by qRT-PCR assay (**A**). The *ARHGDIA* promoter constructs were transfected into PC3 cells, and luciferase activity was measured (**B**). PC3 cells were treated with actinomycin D (5 µg/mL) for 2 h, followed by measurement of *ARHGDIA* mRNA levels at indicated times (**C**). Protein levels of ELAVL1, NCL, and HNRNPD were determined by western blotting and quantitatively analyzed (**D, E**). Data were presented as means ± SEM (n = 3), * *p* < 0.05 vs. the control cells. **F**-**G,** Immumohistochemical staining was performed to evaluate the expression of ELAVL1 in 25 paired human PCa tissues and their adjacent normal prostate tissues (**F**), and ELAVL1 protein levels (**G**) were analyzed by calculating the integrated optical density per area (IOD/area). Data were presented as means ± SEM (n = 25), *** *p* < 0.001. **H,** Correlation between *ELAVL1* mRNA expression and survival of PCa patients was analyzed using Kaplan-Meier survival curve analysis methods based on the TCGA database. **I**-**K,** PC3 cells were transfected with sh-*ELAVL1*. Protein levels of ELAVL1 and ARHGDIA were determined by western blotting (**I**). The migration and invasion abilities of indicated cells were evaluated. Representative images (**J**) and quantification of the cell migration and invasion assay results were shown (**K**). Data were presented as means ± SEM (n = 5), * *p* < 0.05 vs. the control cells. **L**-**P,** PCa cells were transfected with sh-*METTL3* before transfection with sh-*ELAVL1*. Protein levels of METTL3, ELAVL1, and ARHGDIA were determined by western blotting and quantitatively analyzed (**L, M**). The migration and invasion abilities of indicated cells were evaluated. Representative images and quantification of the cell migration and invasion assay results were shown (**N, O**). PC3 cells were treated with actinomycin D (5 µg/mL) for 2 h, followed by measurement of *ARHGDIA* mRNA levels at indicated times (**P**). Data were presented as means ± SEM (n = 3), * *p* < 0.05 vs. the control cells, # *p* < 0.05 vs. the sh-*METTL3*-treated cells. **Q,** ELAVL1 was immunoprecipitated, followed by qRT-PCR assay to evaluate the association of the *ARHGDIA* transcripts with ELAVL1 protein. Data were presented as means ± SEM (n = 3), * *p* < 0.05.

We found that *ARHGDIA* mRNA levesl were markedly reduced in response to METTL3 knockdown **(Figure 4A and Figure S6A)**. Moreover, comparable *ARHGDIA* promoter transcriptional activity between METTL3 knockdown cells and control cells were examined, excluding the possibility that METTL3 downregulation inhibited *ARHGDIA* transcription **(Figure 4B and Figure S6B)**. We then investigated the possibility that METTL3 knockdown impaired the stability of *ARHGDIA* mRNA. As expected, we observed elevated decay rates of *ARHGDIA* mRNA upon the knock down of METTL3 **(Figure 4C and Figure S6C)**.

To determine the upstream regulators mediating *ARHGDIA* mRNA stabilization by METTL3, we examined expression of hetergeneous nuclear ribonucleoprotein D (HNRNPD), ELAV like RNA-binding protein 1 (ELAV1), and nucleolin (NCL), which were identified to bind to mRNA and regulated mRNA stability. The results showed that ELAVL1 was upregulated in PCa cells after knock down of METTL3 **(Figure 4D**-**E and Figure S6D**-**E)**. Accordingly, we determined reduced ELAVL1 expression in human PCa tissues compared to normal prostate tissues **(Figure 4F**-**G)**. It was noted that no significant correlation of ELAVL1 mRNA levels with survival of PCa patients were observed** (Figure 4H)**.

We observed that ELAVL1 knockdown markedly increased ARHGDIA expression and promoted migration and invasion of PCa cells **(Figure 4I**-**K and Figure S6F**-**H)**. Additionally, the knockdown of ELAVL1 effectively attenuated METTL3 knockdwon-induced ARHGDIA reduction and concurrent imparied invasion ability **(Figure 4L**-**O and Figure S6I**-**L)**. Consistently, ELAVL1 knockdown markedly slowed down the decay rates of *ARHGDIA* mRNA **(Figure 4P and Figure S6M)**. As expected, METTL3 knockdown significantly enhanced the interaction between ELAVL1 and *ARHGDIA* mRNA, and the interaction was significantly inhibited upon knock down of ELAVL1 **(Figure 4Q and Figure S6N)**.

### METTL3 induces degradation of ELAVL1 protein by reducing USP4 protein levels

**Figure 5:**
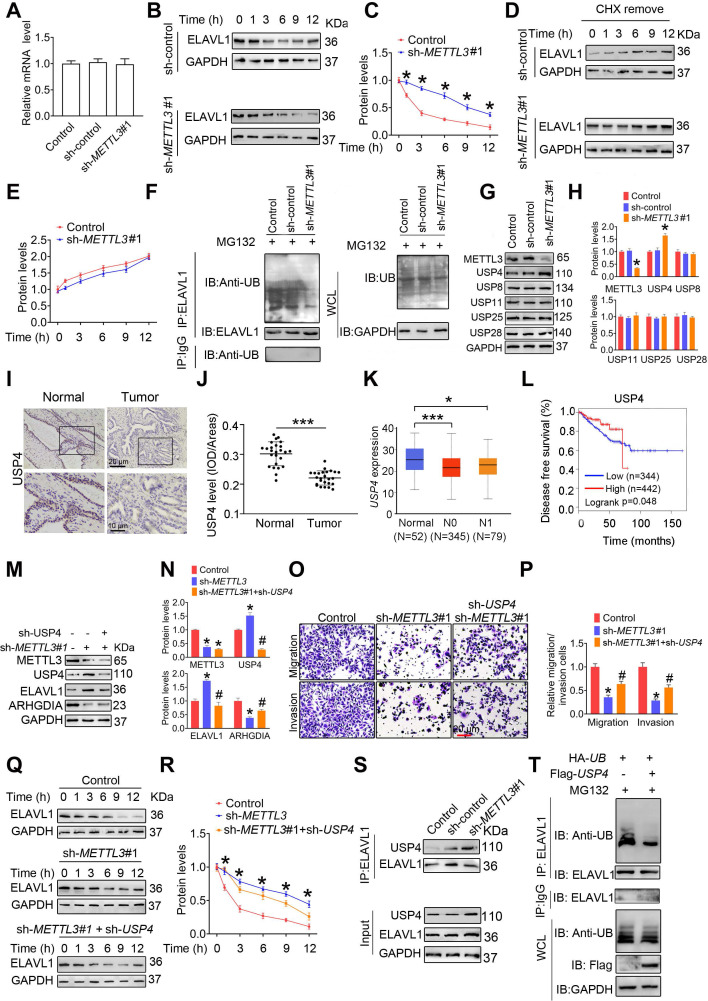
** USP4 is an METTL3 downstream effector and mediates ELAVL1 protein stability. A,** The *ELAVL1* mRNA levels in indicated cells were determined by qRT-PCR assay. **B**-**C,** The indicated PC3 cells were pretreated with cycloheximide (CHX, 10 µg/mL) for 3 h, followed by measurement of ELAVL1 protein levels at indicated times. **D**-**E,** PC3 cells were treated with cycloheximide (CHX) for 12 h. After washing out CHX, cells were cultured for the indicated times. ELAVL1 synthesis levels were determined by western blotting and quantitatively analyzed. **F,** PC3 cells were treated with MG132 for 6 h. Lysates from the indicated cells were subjected to coimmunoprecipitation (Co-IP) assay with anti-ELAVL1 antibody, and the blots were then probed with anti-ubiquitin (UB) antibody for detection of ubiquitination of ELAVL1. **G**-**H,** Protein levels of ELAVL1, NCL, and HNRNPD in indicated cells were determined by western blotting and quantitatively analyzed. Data were presented as means ± SEM (n = 25), * *p* < 0.001 vs. the control cells. **I**-**J,** Immumohistochemical staining was performed to evaluate the expression of USP4 in 25 paired human PCa tissues and their adjacent normal prostate tissues (**I**), and USP4 protein levels (**J**) were analyzed by calculating the integrated optical density per area (IOD/area). Data were presented as means ± SEM (n = 25), *** *p* < 0.001. **K,** The *USP4* mRNA levels were analyzed in the TCGA database. **L,** Correlation between *USP4* mRNA expression and survival of PCa patients was analyzed using Kaplan-Meier survival curve analysis methods based on the TCGA database. **M**-**R,** PC3 cells were transfected with sh-*METTL3* before transfection with sh-*ELAVL1*. Protein levels of METTL3, USP4, ELAVL1, and ARHGDIA were determined by western blotting and quantitatively analyzed. The migration and invasion abilities of indicated cells were evaluated. Representative images and quantification of the cell migration and invasion assay results were shown. The indicated cells were pretreated with CHX for 3 h, followed by measurement of ELAVL1 protein levels at indicated times. **S,** Lysates from the indicated cells were subjected to Co-IP with anti-ELAVL1, and the blots were probed with anti-USP4 antibody. **T,** PC3 cells were transfected with indicated plasmids, and ubiquitination of ELAVL1 was measured by Co-IP assay. Data were presented as means ± SEM (n = 3), * *p* < 0.05 vs. the control cells, # *p* < 0.05 vs. the sh-*METTL3*-treated cells. WCL: whole cell lystate.

In this study, we examined that METTL3 knockdown did not affect ELAVL1 mRNA levels in PCa cells **(Figure 5A and Figure S7A)**, consistent with the survival analyses results that no significant association of *ELAVL1* mRNA levels with survival rate of PCa patients were examined **(Figure 4H)**. Thus, we speculated that the increase of protein stability or enhancement of translation efficiency might upregulate ELAVL1 protein levels in PCa cells upon METTL3 knockdown. Moreover, we observed that degradation rate of ELAVL1 was markedly attenuated in PCa cells in response to METTL3 knockdown **(Figure 5B**-**C and Figure S7B**-**C)**, while no significant changes of synthesis rate of ELAVL1 was observed **(Figure 5D**-**E and Figure S7D**-**E)**. Taken together, these results lead us to believe that METTL3 might reduce ELAVL1 protein levels by inducing its degradation.

To investigate whether ubiquitin-proteasome pathway participates in ELAVL1 degradation, we firstly assessed the effect of METTL3 knockdown on ubiquitination levels of ELAVL1. We found that knocking down METTL3 remarkably reduced the ubiquitination level of ELAVL1 **(Figure 5F and Figure S7F)**. Given that protein deubiquitination is regulated by deubiquitinating enzymes (DUBs) and ubiquitin-specific protease (USP) family is the largest family of DUBs 28, 29, we then compared the mRNA levels of a total of 42 USP members by analyzing the TCGA data **(Table S7)**. We found that *USP4*, *USP8*, *USP11*, *USP25* and *UPS28* mRNA levels were markedly decreased in PCa tissues in comparion to normal prostate tissues, while *USP3*, *USP7*, *USP22* and *USP40* mRNA levels were increased. Based on the fact that METTL3 knockdown decrease ubiquitination level of ELAVL1 and METTL3 protein levels were upregulated in PCa tissue, we speculate that expression of the target USP members involving ELAVL1 regulation might be decreased in PCa tissues. And thus, we chose USP4, USP8, USP11, USP25 and USP28 for further analyses, and western blotting revealled that METTL3 knockdown markedly increased USP4 protein levels **(Figure 5G**-**H and Figure S7G**-**H)**. Moreover, we determined reduced USP4 levels in human PCa tissues relative to normal prostate tissues** (Figure 5I**-**J)**. Consistently, a poorer survival rate was observed to be associated with lower USP4 mRNA levels **(Figure 5K**-**L)**.

Further studies identified that the USP4 knockdown effectively attenuated METTL3 knockdown-induced ELAVL1 increase, ARHGDIA reduction, and concurrent inhibited migratory and invasive capacities of PCa cells **(Figure 5M**-**P and Figure S7I**-**K)**. Consistently, USP4 knockdown markedly reduced protein stability of ELAVL1 **(Figure 5Q**-**R and Figure S7L**-**M)**.

To understand the molecular mechanism accounting for the regulation of ELAVL1 by USP4, we firsty assesed the interaction beween USP4 and ELAVL1 in PCa cells. The results showed that METL3 knockdown markedly enhanced the interaction between USP4 and ELAVL1 **(Figure 5S and Figure S7N)**. Additionally, the overexpression of USP4 effectively decreased the ubiquitination levels of ELAVL1 **(Figure 5T and Figure S7O)**. Taken together, our results demonstrated that USP4 upregulates ELAVL1 expression by removing the ubiquitin group from ELAVL1 protein.

### m^6^A methylation reduces USP4 protein levels by affecing *USP4* mRNA stability

**Figure 6:**
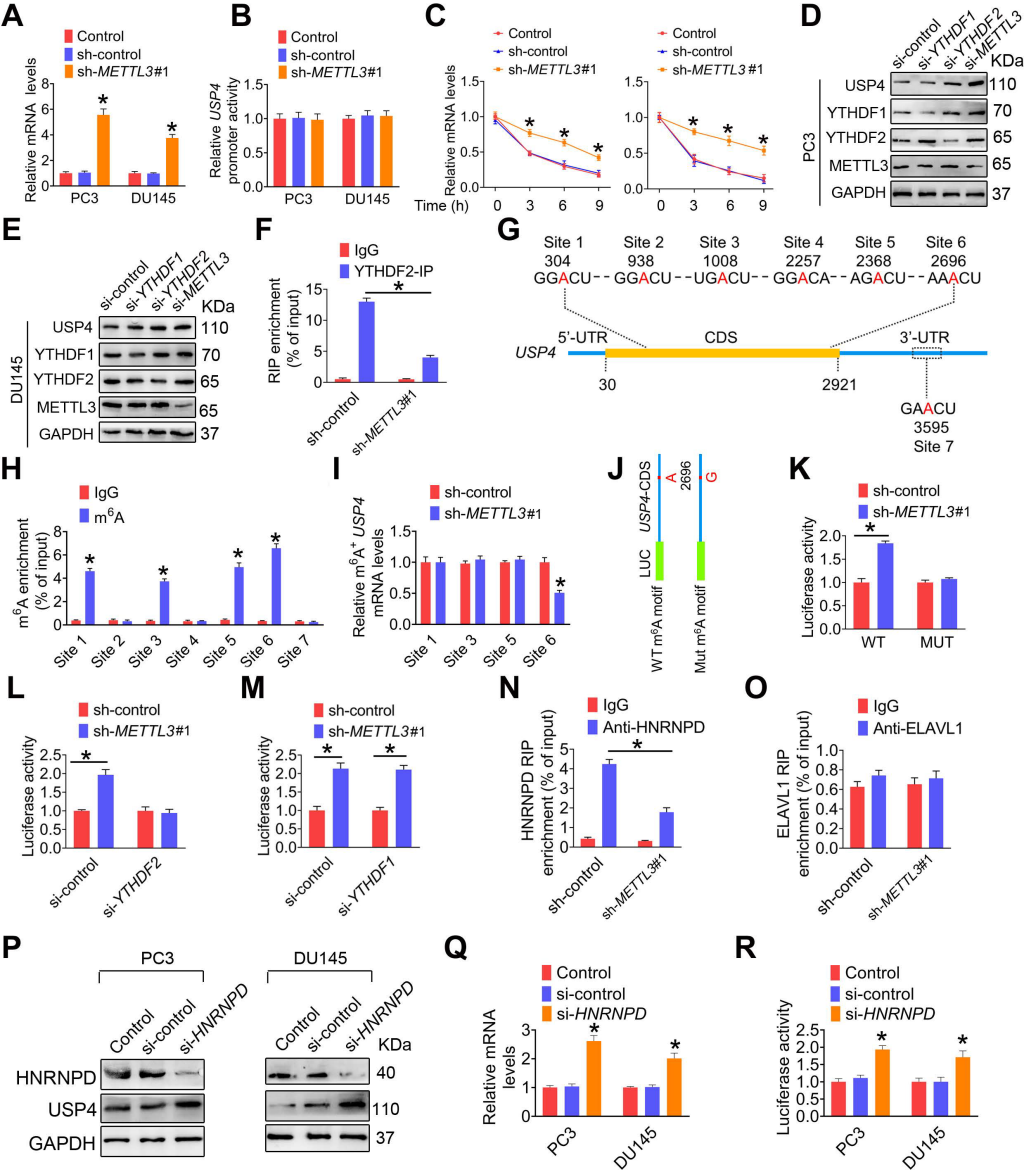
**m^6^A induces decay of the USP4 transcript in prostate cancer (PCa) cell. A**-**C,** PC3 and DU145 cells were transfected with shRNA targeting *METTL3* (sh-*METTL3*), respectively. The *USP4* mRNA levels in indicated cells were determined by qRT-PCR assay (**A**). The *USP4* promoter constructs were transfected into indicated cells, and luciferase activity was measured (**B**). PCa cells were treated with actinomycin D (5 μg/mL) for 2 h, followed by measurement of *USP4* mRNA levels at indicated times (**C**). Data were presented as means ± SEM (n = 3), * *p* < 0.05 vs. the control cells. **D**-**E,** PC3 cells (**D**) and DU145 cells (**E**) were treated with indicated siRNAs, and cell lysates were subject to western blotting. **F,** Lysates from the PC3 cells were subjected to immunoprecipitation with anti-YTHDF2, and the association of the USP4 transcript with YTHDF2 was determined by qRT-PCR. Data were presented as means ± SEM (n = 3), * *p* < 0.05. **G,** Schematic representation of the position of m^6^A motifs within *USP4* transcript. **H,** Abundance of USP4 transcript among mRNA immunoprecipitated with anti-m^6^A antibody was measured by qRT-PCR and normalized to input. Data are presented as means ± SEM (n = 3). * *p* < 0.05 vs. the IgG group. **I,** Abundance of *USP4* transcript among mRNA immunoprecipitated with anti-m^6^A antibody was measured by qRT-PCR. Data are presented as means ± SEM (n = 3). * *p* < 0.05 vs. the control cells. **J,** USP4-CDS of the wild-type or mutant (A to G) was fused with a luciferase reporter. **K**-**M,** Luciferase activity of USP4-CDS was measured and normalized to Renilla luciferase activity. Data are presented as means ± SEM (n = 3). * *p* < 0.05. **N**-**O,** Lysates from the indicated cells were subjected to immunoprecipitation with anti-HNRNPD (**N**) or anti-ELAVL1 (**O**), and the association of the* USP4* transcript with each protein was determined by qRT-PCR. Data were presented as means ± SEM (n = 3), * *p* < 0.05. **P**-**R,** PC3 cells and DU145 cells were treated with indicated siRNAs, and cell lysates were subject to western blotting (**P**). The *USP4* mRNA levels in indicated cells were determined by qRT-PCR (**Q**). Luciferase activity of USP4-CDS in indicated cells was measured and normalized to Renilla luciferase activity (**R**). Data are presented as means ± SEM (n = 3), * *p* < 0.05 vs. the control cells.

We next found that METTL3 knockdown significantly upregulated *USP4* mRNA levels in PCa cells **(Figure 6A)**. Moreover, *USP4* promoter transcriptional activity was not affected upon METTL3 knockdown **(Figure 6B)**, excluding the possibility that METTL3 downregulation inhibited *USP4* transcription. By contrast, we observed that knock down of METTL3 slowed down degradation rate of *USP4* mRNA** (Figure 6C)**. Given the key role of m^6^A mRNA methylation in affecting mRNA stability, it was reasonable to speculate that METTL3 knockdown reduces the m^6^A modification levels in* USP4* mRNA and thus increase *USP4* mRNA levels by atenuating the inhibitory effect of m^6^A modification on *USP4* mRNA stability. Consistent with this hypothesis, it was knockdown of YTHDF2 but not YTHDF1 remarkably upregulated USP4 protein levels to a simlar extent as METTL3 knockdown in PCa cells **(Figure 6D**-**E)**. Furthermore, YTHDF2 enrichment at *USP4* transcripts was markedly reduced in PC3 cells upon knockdown of METTL3** (Figure 6F and Figure S8A)**.

We next performed sequence anlysis of *USP4* transcript and found six m^6^A modification sites within the CDS region and one m^6^A site in the 3'-UTR** (Figure 6G)**. m^6^A RIP analyses demonstrated that m^6^A was markedly enriched at site 1, site 3, site 5, and site 6 **(Figure 6H)**. Moreover, m^6^A enrichment levels at site 6 were significantly reduced upon knockdown of METTL3 **(Figure 6I)**. We generated a luciferase reporter construct containing a firefly luciferase placed before the *USP4*-CDS **(Figure 6J)**, and subsequent luciferase reporter assays demonstrated that METTL3 knockdown significantly increased the reporter luciferase activity **(Figure 6K)**; however, knock down of YTHDF2 but not YTHDF1 effectivelly aborted this increase** (Figure 6L**-**M)**. Additionally, we mutated the potential m^6^A motif AAACU to AAGCU, and METTL3 knockdown cannot upregulate the luciferase activity of the reporter bearing mutated* USP4*-CDS** (Figure 6K)**.

It has been reported that HNRNPD and ELAVL1 could bind to m^6^A-modified transcript to regulate pre-mRNA splicing or affect mRNA stability 10, 30. In this study, we examined that METTL3 knockdown significantly decreased the binding of HNRNPD to *USP4* pre-mRNA **(Figure 6N and Figure S8B)**; however, the interaction between ELAVL1 and *USP4* pre-mRNA were not significantly changed** (Figure 6O and Figure S8C)**. Moreover, HNRNPD deficiency significantly increased *USP4* expression at mRNA and protein levels, accompanied by a increase of *USP4*-CDS luciferase activity **(Figure 6P**-**R)**. Taken together, these results suggested m^6^A methylation reduces *USP4* mRNA stability by promoting the binding of HNRNPD to* USP4* mRNA.

### Dysregulation of MELLT3-USP4-ELAVL1-ARHGDIA regulatory axis hinders PCa metastasis

**Figure 7:**
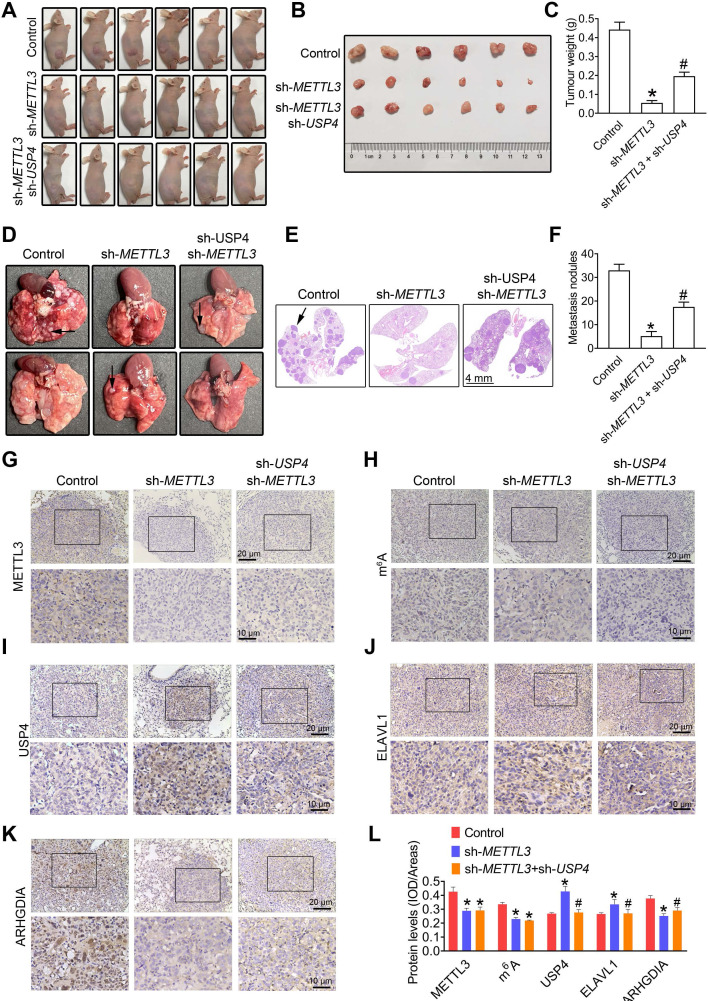
** METTL3-USP4-ELAVL1-ARHGDIA regulation axis promotes prostate cancer (PCa) metastasis.** PC3 cells were transfected with shRNA targeting *METTL3* (sh-*METTL3*) before transfection with sh-*USP4*. **A**-**C,** Athymic nude mice were subcutaneously injected into the right axillary region of each mouse with indicated cells. Eight weeks after cell injection, the mice were sacrificed and the tumors were surgically removed and photographed (**A, B**), as well as weighed (**C**). D-F, The PC3 cells were injected into the SCID mice by tail vein injection. Representative images of metastatic nodules in the lung (**D**) and the H&E staining results were shown (**E**), and the number of metastatic nodules were quantitatively analyzed (**F**).** G**-**K,** The lung tissues obtained from SCID mice were subjected to immumohistochemical staining assay for evaluating METTL3 (**G**), m^6^A modification (**H**), USP4 (**I**), ELAVL1 (**J**), and ARHGDIA (**K**) levels. **L,** The m^6^A levels and the expression levels of the target proteins were analyzed by calculating the integrated optical density per area (IOD/area). Data were presented as means ± SEM (n = 6), * *p* < 0.05 vs. the control cells, # *p* < 0.05 vs. the sh-*METTL3*-treated cells.

To further confirm the role of METTL3-ELAVL1-ARHGDIA regulatory axis in regulating PCa metastasis, we examined expression of downstream effector of METTL3 including USP4, ELAVL1, and ARHGDIA in those PCa cells with *METTL3* overexpression. As predicted, we found that METTL3 overexpression induced ARHGDIA expression, but downregulated ELAVL1 and USP4 **(Figure S9)**. We noted that METTL3 knockdown markedly suppressed xenograft tumor formation and reduced the tumor weight **(Figure 7A**-**C)**, consistent with the cell cycle assay results demonstrating that METTL3 knockdown exerted inhibitory effects on cell proliferation of PC3 cells *in vitro* **(Figure S10)***.* Moreover, we observed that USP4 knockdown effectively alleviated METTL3 knockdown-induced inhibited xenograft tumor growth** (Figure 7A**-**C)**. To further confirm the role of METTL3-ELAVL1- ARHGDIA regulatory axis in regulating PCa metastasis, we also examined expression of USP4, ELAVL1 and ARHGDIA in xenograft model with METTL3-deficient PCa cells. We observed reduced METTL3, m^6^A, and ARHGDIA levels, but increased USP4 and ELAVL1 levels in* in vivo* xenograft nude mice injected with those PC3 cells with METTL3 knock down **(Figure S11)**; however, the knockdown of USP4 effectivly attenuated METTL3 deficiency-induced these changes **(Figure S11)**.

Moreover, METTL3 silencing dramatically suppressed lung metastatic abilities of PC3 cells, as evidenced by the number of lung metastatic nodules **(Figure 7D**-**F)**; however, the knockdown of USP4 effectively attenuated METTL3 deficiency- induced decreased lung metastatic capacity of PC3 cells **(Figure 7D**-**F)**. As expected, we observed a reducation in ARHGDIA protein levels, and USP4 and ELAVL1 increase in lung metastatic lesions of sh-*METTL3* cells-injected mice, accompanied with METTL3 and m^6^A decrease **(Figure 7G**-**L)**, and USP4 knock down effectivelly reversed METTL3 deficiency-induced these changes.

### Reduced promoter methylation is associated with increased expression of METTL3

**Figure 8:**
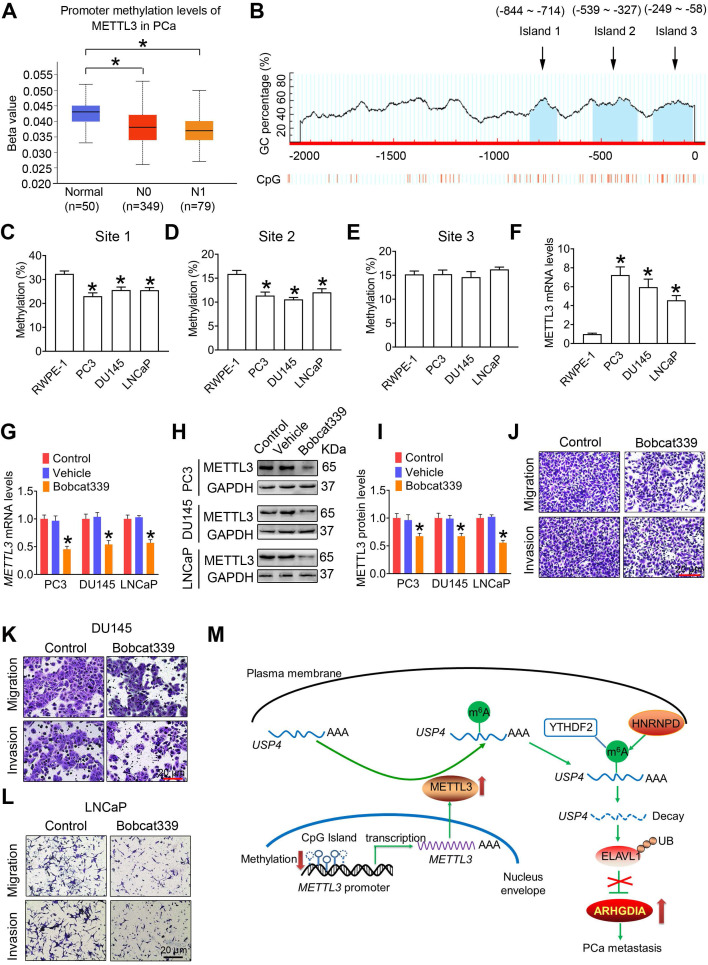
** Enhanced METTL3 expression is associated with reduced *METTL3* promoter methylation in prostate cancer (PCa) cells. A,** The methylation levels of *METTL3* promoter were analyzed based on the TCGA database (*p < 0.05). **B,** Potential CpG islands in the human *METTL3* promoter were analyzed using online MethPrimer software (http://www.urogene.org/methprimer/), and the blue shaded regions indicate the potential CpG islands. **C**-**E,** The methylation status of the *USP4* promoter at different CpG islands was measured by methylation-specific PCR. Data were presented as means ± SEM (n = 5), * *p* < 0.05 vs. the RWPE-1 cells. **F,** Celluarl *METTL3* mRNA levels were determined by qRT-PCR. Data were presented as means ± SEM (n = 5), * *p* < 0.05 vs. the RWPE-1 cells. **G**-**L,** PCa cells were treated with Bobcat339 at 50 μM for 48 h. The *METTL3* mRNA levels in PCa cells were determined by qRT-PCR (**G**). The METTL3 protein levels in PCa cells were determined by western blotting and quantitatively analyzed (**H, I**). The migration and invasion abilities of indicated cells were evaluated, and representative images were shown (**J-L**). Data were presented as means ± SEM (n = 3), * *p* < 0.05 vs. the control cells. **M,** A model for the critical link between METTL3 and PCa metastasis is proposed. Reduced *METTL3* promoter methylation increases METTL3 expression by promoting its transcription. Upregulation of METTL3 increases cellular m^6^A mRNA methylation levels, which downregulates USP4 expression by inducing m^6^A-mediated decay of the* USP4* transcript. USP4 reduction leads to decreased expression of ELAVL1 by increasing ubiquitination (UB) of ELAVL1, contributing to upregulated expression of ARHGDIA by retarding ELAVL1-mediated decay of the *ARHGDIA* transcript.

We found that methylation levels in the METTL3 promoter were significantly reduced in PCa tissues comparted to the adjoining normal prostate tissues by analyzing the TCGA database **(Figure 8A)**. We next bioinformatically analyzed the potential CpG island of human *METTL3* promoter, and a total of three CpG island signals were predicted in the *METTL3* promoter region **(Figure 8B)**. Additionally, we found that the methylation levels at site 1 and site 2 were significantly reduced in PCa cells relative to the RWPE-1 cells, accompanied by increased *METTL3* mRNA levels in PCa cells** (Figure 8C**-**F)**.

It is noted that DNA methylation is dynamically regulated by methyl writing enzymes and methyl erasing enzymes. In this study, we examined that *METTL3* mRNA and protein levels were significantly downregulated in PCa cells following exposure to Bobcat339, the inhibitor of methyl erasing enzymes **(Figure 8G**-**I)**. Accordingly, Bobcat339 effectively decreased migratroy and invasive capacitivities of PCa cells **(Figure 8J**-**L)**.

## Discussion

Growing evidences have suggested that m^6^A modifications play important roles in mediating progression of various cancers, including hepatocellular carcinoma 31, gastric cancer 12, lung cancer 15, endometrial cancer 13, nasopharyngeal cancer 32, bladder cancer 33, and acute myeloid leukaemia 34. Moreover, METTL3 has been reported to be upregulated or downregulated in certain cancers, and its specific roles in tumorigenesis remain controversial. Recently, Lin et al. observed upregulated METTL3 levels in lung adenocarcinoma and identified that METTL3 promotes growth of lung cancer cells 15. By contrast, Liu et al. determined decreased METTL3 expression and concurrent reduction in m^6^A methylation levels in endometrial tumors, which in turn promotes proliferation of endometrial cancer cells 13. In this study, we determined upregulated expression of METTL3 in PCa tissues, contributing to an increase of m^6^A modification levels. Moreover, we observed that METTL3 knockdown did not change FTO, ALKBH5, and METTL4 levels, excluding the possibility that METTL3 silencing firstly affects expression of other m^6^A-modification related proteins and then reduce m^6^A modification levels of downstream targets. Further studies identified that reduced methylation levels at the CpG islands in promoter might be associated with enhanced *METTL3* transcription in PCa cells, and to confirm which DNA methyltransferase are involved in* METTL3* regulation deserves a separate study.

Accordingly, some researchers have also identified that m^6^A modification is associated with growth and migration of PCa cells 35, 36. Cai et al. detected upregulated METTL3 levels in PCa cells, and identified that METTL3 promotes PCa growth by regulating hedgehog pathway 35. Li et al. examined increased YTHDF2 and METTL3 in PCa cells, and proposed that YTHDF2 reduces expression of the tumor suppressors via mediating degradation of the m^6^A-modified mRNAs to induce AKT phosphorylation and subsequent tumor progressionin PCa 37. Ma et al. found that METTL3 activated the Wnt pathway, thereafter, bringing about enhanced migratory ability of the PCa cells 36. It is notedworthy these studies are mainly concentrated on the the role of METTL3 in regulating upstream signaling pathways associated with PCa progression, and we found that METTL3 directly affect expression of migration-related key protein ARHGDIA to influence PCa migration and invasion. Based on these results, we speculate that this type of multiayed regulation of METTL3 in downstream proteins and upstream signaling pathways might effectively mediate PCa progression; however, whether the Hedgehog, Akt, and Wnt signaling pathways are involved in METTL3-mediated ARHGDIA expression deserves a separate study. Moreover, these researchers did not validate their findings simultaneously in many PCa cell lines, subcutaneous xenografts of PCa in nude mice, and clinical samples from patients diagnosed with PCa. Compared with these researchers, we also compared the differential expression of METTL3 in androgen-dependent and androgen-independent PCa cells and confirmed the effects of androgen on the METTL3 expression in androgen-dependent LNCaP cells. In this study, we examined higher METTL3 levels in PC3 and DU145 PCa cells than LNCaP cells, which may be associated with deadly metastatic capacity of androgen-independent PCa cells. Moreover, testosterone significantly induced METTL3 expression in LNCaP cells but not PC3 and DU145 cells, indicating that androgen might modulates PCa metastasis by upregulating METTL3 expression and m^6^A levels after binding to AR in PCa cells.

EMT and migration of cancer cells are two important biological phenomenon during the tumorigenic process. In this study, we found that METTL3 knockdown did not affect expression of EMT-related genes, whereas significantly reduced migration-related ARHGDIA expression. ARHGDIA has been reported to modulate several processes during tumorigenesis, including cellular growth, cellular migration and cellular polarity. Up to date, Zhu, et al. has demonstrated that ARHGDIA upregulation inhibits growth of PCa cells, while ARHGDIA deficiency markedly promote the growth of androgen-sensitive LNCaP cells in androgen-deprived conditions 38. Yamashita, et al. observed upregulated ARHGDIA levels in lymph node metastatic PCa patients and suggested that ARHGDIA may be useful as a diagnostic biomarker for PCa metastasis 23. To sum up, the role of ARHGDIA in PCa progression remains elusive. In the present study, we observed an increase of ARHGDIA protein levels in human PCa tissues compared with the normal prostate tissues. Moreover, ectopic expression of ARHGDIA effectively attenuated the effect of METTL3 knockdown on invasive ability in PCa cells. Based on these results, we believe that METTL3 could promote PCa metastasis by promoting ARHGDIA expression. Further studies identified that METTL3 stabilited *ARHGDIA* mRNA by modulating expression of the mRNA binding protein ELAVL1, which in turn alleviated decay of *ARHGDIA* mRNA. Interestinly, it is noted that ELAVL1 can bind to and stabilize AU-rich element-mRNAs 39, and thus we speculate there are other factors involved in METTL3-mediated degradation of *ARHGDIA* mRNA, which deserves a follow-up study.

Subsequent studies revealled that USP4 was upregulated in PCa cells upon METTL3 knockdown. Some researchers found that USP4 was markedly increased and verified to play a tumor-promoting role in liver cancer 40, 41 and colorectal cancer 42, 43. By contrast, USP4 was reported to play a tumor-suppressing role in breast cancer 44 and lung cancer 45. Interestingly, USP4 may exert completely opposite effects even in the same tumor due to its different upstream or downstream effectors 46, 47. Based on these results, we speculated that the specific roles of USP4 in modulating cancer progression are complicated and diverse, which depend on tumor types and tumor microenvironment. In this study, we provided complelling evidence to identify that METTL3 modulates PCa metastasis. Moreover, we also found that the knockdown of USP4 effectively alleviated METTL3 decifiency-induced suppressed xenograft tumor formation. These results suggested that USP4, as an downstream target of METTL3, might also regulate PCa growth, which deserves a follow-up study.

By performing m^6^A RIP-qRT-PCR assay, we found that USP4 was the key downstream target of METTL3 in PCa progression. Subsequent studies demonstrated that m^6^A could decrease USP4 expression by inducing m^6^A-dependent decay of the *USP4* transcript. Moreover, we identified that METTL3 could methylate USP4 at one m^6^A site within the CDS and around the stop codon, and the m^6^A reader protein YTHDF2 could recognize the m^6^A site and then recruit RNA-binding protein HNRNPD to the mRNA, resulting in degradation of *USP4* mRNA.

## Conclusions

Collectively, we have provided complelling evidence to identify that METTL3 promotes PCa metastasis by upregulating ARHGDIA expression (Figure 8M). These results presented here suggested that METTL3 might be a favorable predictor for PCa, and this study also provides insight into novel therapeutic strategies by inhibiting METTL3 expression for suppressing PCa metastasis.

## Supplementary Material

Supplementary figures and tables.

## Abbreviations

ALKBH5: alkB homolog 5
FTO: fat mass and obesity-associated protein
m^6^A: N6-methyladenosine
METTL3: Methyltransferase like 3
PCa: Prostate cancer
RIP: RNA-immunoprecipitation
qRT-PCR: quantitative real-time PCR
TCGA: The Cancer Genome Atlas
WTAP: Wilms tumor 1-associating protein
YTHDF2: YTH-domain family 2

## References

[1] Ju LG, Zhu Y, Long QY, Li XJ, Lin X, Tang SB (2019). SPOP suppresses prostate cancer through regulation of CYCLIN E1 stability. Cell Death Differ.

[2] Shin SH, Lee GY, Lee M, Kang J, Shin HW, Chun YS (2018). Aberrant expression of CITED2 promotes prostate cancer metastasis by activating the nucleolin-AKT pathway. Nat Commun.

[3] Eggener SE, Scardino PT, Walsh PC, Han M, Partin AW, Trock BJ (2011). Predicting 15-year prostate cancer specific mortality after radical prostatectomy. J Urol.

[4] Siegel RL, Miller KD, Jemal A (2017). Cancer Statistics, 2017. CA Cancer J Clin.

[5] Deep G, Jain AK, Ramteke A, Ting H, Vijendra KC, Gangar SC (2014). SNAI1 is critical for the aggressiveness of prostate cancer cells with low E-cadherin. Mol Cancer.

[6] Singh R, Karri D, Shen H, Shao JY, Dasgupta S, Huang SX (2018). TRAF4-mediated ubiquitination of NGF receptor TrkA regulates prostate cancer metastasis. J Clin Invest.

[7] Yuan H, Han Y, Wang X, Li N, Liu Q, Yin Y (2020). SETD2 Restricts Prostate Cancer Metastasis by Integrating EZH2 and AMPK Signaling Pathways. Cancer cell.

[8] Zhang SC, Zhao BX, Zhou AD, Lin KY, Zheng SP, Lu Zhike (2017). The m6A Demethylase ALKBH5 Maintains Tumorigenicity ofGlioblastoma Stem-Like Cells by Sustaining FOXM1 Expression and CellProliferation Program. Cancer Cell.

[9] Shi H, Wei J, He C (2019). Where, When, and How: Context-Dependent Functions of RNA Methylation Writers, Readers, and Erasers. Mol Cell.

[10] Song H, Feng X, Zhang H, Luo Y, Huang J, Lin M (2019). METTL3 and ALKBH5 oppositely regulate m(6)A modification of TFEB mRNA, which dictates the fate of hypoxia/reoxygenation-treated cardiomyocytes. Autophagy.

[11] Zhang S, Zhao BS, Zhou A, Lin K, Zheng S, Lu Z (2017). m(6)A Demethylase ALKBH5 Maintains Tumorigenicity of Glioblastoma Stem-like Cells by Sustaining FOXM1 Expression and Cell Proliferation Program. Cancer cell.

[12] Wang Q, Chen C, Ding Q, Zhao Y, Wang Z, Chen J (2020). METTL3-mediated m(6)A modification of HDGF mRNA promotes gastric cancer progression and has prognostic significance. Gut.

[13] Liu J, Eckert MA, Harada BT, Liu SM, Lu Z, Yu K (2018). m(6)A mRNA methylation regulates AKT activity to promote the proliferation and tumorigenicity of endometrial cancer. Nat Cell Biol.

[14] Chen Y, Wang J, Pan C, Li D, Han X (2018). Microcystin-leucine-arginine causes blood-testis barrier disruption and degradation of occludin mediated by matrix metalloproteinase-8. Cell Mol Life Sci.

[15] Lin S, Choe J, Du P, Triboulet R, Gregory RI (2016). The m(6)A Methyltransferase METTL3 promotes Translation in Human Cancer Cells. Mol Cell.

[16] Chen Y, Wang J, Xu D, Xiang Z, Ding J, Yang X (2020). m(6)A mRNA methylation regulates testosterone synthesis through modulating autophagy in Leydig cells. Autophagy.

[17] Chen M, Wei L, Law CT, Tsang FH, Shen J, Cheng CL (2018). RNA N6-methyladenosine methyltransferase-like 3 promotes liver cancer progression through YTHDF2-dependent posttranscriptional silencing of SOCS2. Hepatology.

[18] Taylor BS, Schultz N, Hieronymus H, Gopalan A, Xiao Y, Carver BS (2010). Integrative genomic profiling of human prostate cancer. Cancer Cell.

[19] Varambally S, Yu J, Laxman B, Rhodes DR, Mehra R, Tomlins SA (2005). Integrative genomic and proteomic analysis of prostate cancer reveals signatures of metastatic progression. Cancer Cell.

[20] Grasso CS, Wu YM, Robinson DR, Cao X, Dhanasekaran SM, Khan AP (2012). The mutational landscape of lethal castration-resistant prostate cancer. Nature.

[21] Chandrashekar DS, Bashel B, Balasubramanya SAH, Creighton CJ, Ponce-Rodriguez I, Chakravarthi B (2017). UALCAN: A Portal for Facilitating Tumor Subgroup Gene Expression and Survival Analyses. Neoplasia.

[22] Tang Z, Li C, Kang B, Gao G, Li C, Zhang Z (2017). GEPIA: a web server for cancer and normal gene expression profiling and interactive analyses. Nucleic Acids Res.

[23] Yamashita T, Okamura T, Nagano K, Imai S, Abe Y, Nabeshi H (2012). Rho GDP-dissociation inhibitor alpha is associated with cancer metastasis in colon and prostate cancer. Pharmazie.

[24] Zhu JL, Tian ZX, Li Y, Hua XH, Zhang DY, Li JX (2019). ATG7 Promotes Bladder Cancer Invasion via Autophagy-Mediated Increased ARHGDIB mRNA Stability. Adv Sci (Weinh).

[25] Haga RB, Ridley AJ (2016). Rho GTPases: Regulation and roles in cancer cell biology. Small GTPases.

[26] Liu J, Zhang D, Luo W, Yu Y, Yu J, Li J (2011). X-linked inhibitor of apoptosis protein (XIAP) mediates cancer cell motility via Rho GDP dissociation inhibitor (RhoGDI)-dependent regulation of the cytoskeleton. J Bio Chem.

[27] Zhao L, Wang H, Li J, Liu Y, Ding Y (2008). Overexpression of Rho GDP-dissociation inhibitor alpha is associated with tumor progression and poor prognosis of colorectal cancer. J Proteome Res.

[28] Young MJ, Hsu KC, Lin TE, Chang WC, Hung JJ (2019). The role of ubiquitin-specific peptidases in cancer progression. J Biomed Sci.

[29] Yuan T, Yan F, Ying M, Cao J, He Q, Zhu H (2018). Inhibition of Ubiquitin-Specific Proteases as a Novel Anticancer Therapeutic Strategy. Front Pharmacol.

[30] Wang Y, Li Y, Toth JI, Petroski MD, Zhang Z, Zhao JC (2014). N6-methyladenosine modification destabilizes developmental regulators in embryonic stem cells. Nat Cell Biol.

[31] Lin X, Chai G, Wu Y, Li J, Chen F, Liu J (2019). RNA m(6)A methylation regulates the epithelial mesenchymal transition of cancer cells and translation of Snail. Nat Commun.

[32] Zhang P, He Q, Lei Y, Li Y, Wen X, Hong M (2018). m(6)A-mediated ZNF750 repression facilitates nasopharyngeal carcinoma progression. Cell Death Dis.

[33] Cheng M, Sheng L, Gao Q, Xiong Q, Zhang H, Wu M (2019). The m(6)A methyltransferase METTL3 promotes bladder cancer progression via AFF4/NF-κB/MYC signaling network. Oncogene.

[34] Vu LP, Pickering BF, Cheng Y, Zaccara S, Nguyen D, Minuesa G (2017). The N(6)-methyladenosine (m(6)A)-forming enzyme METTL3 controls myeloid differentiation of normal hematopoietic and leukemia cells. Nat Med.

[35] Cai J, Yang F, Zhan H, Situ J, Li W, Mao Y (2019). RNA m(6)A Methyltransferase METTL3 Promotes The Growth Of Prostate Cancer By Regulating Hedgehog Pathway. OncoTargets Ther.

[36] Ma XX, Cao ZG, Zhao SL (2020). m6A methyltransferase METTL3 promotes the progression of prostate cancer via m6A-modified LEF1. Eur Rev Med Pharmacol Sci.

[37] Li J, Xie H, Ying Y, Chen H, Yan H, He L (2020). YTHDF2 mediates the mRNA degradation of the tumor suppressors to induce AKT phosphorylation in N6-methyladenosine-dependent way in prostate cancer. Mol Cancer.

[38] Zhu Y, Tummala R, Liu C, Nadiminty N, Lou W, Evans CP (2012). RhoGDIα suppresses growth and survival of prostate cancer cells. Prostate.

[39] Brennan CM, Steitz JA (2001). HuR and mRNA stability. Cell Mol Life Sci.

[40] Liang Y, Song X, Li Y, Ma T, Su P, Guo R (2019). Targeting the circBMPR2/miR-553/USP4 Axis as a Potent Therapeutic Approach for Breast Cancer. Mol Ther Nucleic Acids.

[41] Qiu C, Liu Y, Mei Y, Zou M, Zhao Z, Ye M (2018). Ubiquitin-specific protease 4 promotes metastasis of hepatocellular carcinoma by increasing TGF-β signaling-induced epithelial-mesenchymal transition. Aging (Albany NY).

[42] Nguyen HH, Kim T, Nguyen T, Hahn MJ, Yun SI, Kim KK (2019). A Selective Inhibitor of Ubiquitin-Specific Protease 4 Suppresses Colorectal Cancer Progression by Regulating β-Catenin Signaling. Cell Physiol Biochem.

[43] Zhao B, Schlesiger C, Masucci MG, Lindsten K (2009). The ubiquitin specific protease 4 (USP4) is a new player in the Wnt signalling pathway. J Cell Mol Med.

[44] Geng N, Li Y, Zhang W, Wang F, Wang X, Jin Z (2020). A PAK5-DNPEP-USP4 axis dictates breast cancer growth and metastasis. Int J Cancer.

[45] Liang L, Fan Y, Cheng J, Cheng D, Zhao Y, Cao B (2013). TAK1 ubiquitination regulates doxorubicin-induced NF-κB activation. Cell Signal.

[46] Cao WH, Liu XP, Meng SL, Gao YW, Wang Y, Ma ZL (2016). USP4 promotes invasion of breast cancer cells via Relaxin/TGF-β1/Smad2/MMP-9 signal. Eur Rev Med Pharmacol Sci.

[47] Li Y, Jiang D, Zhang Q, Liu X, Cai Z (2016). Ubiquitin-specific protease 4 inhibits breast cancer cell growth through the upregulation of PDCD4. Int J Mol Med.

