# Silencing of METTL3 effectively hinders invasion and metastasis of prostate cancer cells: Erratum

**DOI:** 10.7150/thno.105051

**Published:** 2024-11-02

**Authors:** Yabing Chen, Chun Pan, Xiaotong Wang, Dihui Xu, Yuhan Ma, Jianhang Hu, Peilin Chen, Zou Xiang, Qiu Rao, Xiaodong Han

**Affiliations:** 1 Immunology and Reproduction Biology Laboratory & State Key Laboratory of Analytical Chemistry for Life Science, Medical School, Nanjing University, Nanjing 210093, China.; 2 Jiangsu Key Laboratory of Molecular Medicine, Nanjing University, Nanjing 210093, China.; 3 Department of Pathology, Jinling Hospital, Nanjing University School of Medicine, Nanjing 210002, China.; 4 Department of Health Technology and Informatics, Faculty of Health and Social Sciences, The Hong Kong Polytechnic University, Hung Hom, Kowloon, Hong Kong China.

The authors regret that some incorrect representative images were accidentally used in our previously published paper when the first author assembled the figures, including transwell images in Figure 2E, Figure 3K, and Western blot photos in Figure 3A. The correct figures are shown below and in the attached Supporting Information. The authors confirm that these corrections do not change the result interpretation or conclusions of the article. The authors are deeply sorry and sincerely apologize for any inconvenience or misunderstanding that may have caused.

## Figures and Tables

**Figure 2:**
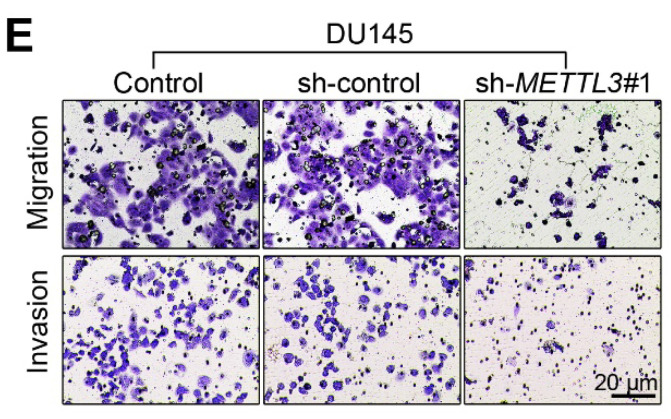
** Corrected image for the original Figure 2E.** Representative images of the cell migration and invasion assay results of DU145 cells were shown.

**Figure 3:**
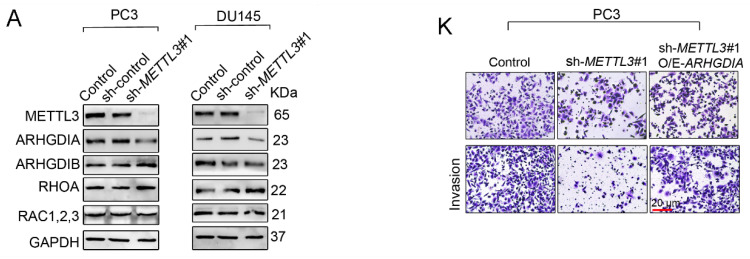
** Corrected image for the original Figure 3A and Figure 3K. A.** Western blotting was used to examine protein expression of invasion- and EMT-related genes. The migration and invasion abilities of PC3 cells were evaluated. **K.** Representative images of the migration and invasion assay results of PC3 cells were shown.

