# Supplementary material for: Silencing of METTL3 effectively hinders invasion and metastasis of prostate cancer cells
Source: Theranostics. 2021 Jun 11;11(16):7640–57. doi: 10.7150/thno.61178 (PMC8315076; doi:10.7150/thno.61178)
Supplement: Supplementary file 1 — Supplementary figures and tables. [file thnov11p7640s1.pdf]

Silencing of METTL3 effectively hinders invasion and metastasis of prostate cancer cells

Yabing Chen[1,2 #], Chun Pan[1,2#], Xiaotong Wang[3], Dihui Xu[1,2], Yuhan Ma[1,2], Jianhang Hu[1,2], Peilin Chen[1,2], Zou Xiang[4], Qiu Rao[3✉], Xiaodong Han[1,2✉]

[1] Immunology and Reproduction Biology Laboratory & State Key Laboratory of Analytical Chemistry for Life Science, Medical School, Nanjing University, Nanjing 210093, China

[2] Jiangsu Key Laboratory of Molecular Medicine, Nanjing University, Nanjing 210093, China

[3] Department of Pathology, Jinling Hospital, Nanjing University School of Medicine, Nanjing 210002, China

[4] Department of Health Technology and Informatics, Faculty of Health and Social Sciences, The Hong Kong Polytechnic University, Hung Hom, Kowloon, Hong Kong China

[#]These authors contributed equally to this study.

✉ Corresponding authors: Qiu Rao, raoqiu1103@126.com, Department of Pathology, Jinling Hospital, Nanjing 210002, China; Xiaodong Han, hanxd@nju.edu.cn, Immunology and Reproduction Biology Laboratory, Medical School, Nanjing University, Nanjing 210093, China, Tel/fax: +86 25 83686497.

Table S1. Primary antibodies used in this study.

Source	Primary antibodies	Catalog no.	Working dilution
Abcam	Rabbit anti-METTL3	ab195352	WB, 1:2000 IHC, 1: 800
Abcam	Mouse anti-FTO	ab92981	WB, 1:1000 IHC, 1:400
Abcam	Rabbit anti-ZEB1	ab203829	WB, 1:500 IHC
Santa Cruz Biotechnology	Mouse anti-ARHGDIB	sc-376473	WB,1:500
Santa Cruz Biotechnology	Mouse anti-E-cadherin	sc-8426	WB,1:500
Santa Cruz Biotechnology	Mouse anti-HNRNPD	sc-166577	WB,1:500
Santa Cruz Biotechnology	Mouse anti-MMP-9	sc-393859	WB,1:500
Santa Cruz Biotechnology	Mouse anti-NCL	sc-17826	WB,1:500
Santa Cruz Biotechnology	Mouse anti-RAC1/2/3	sc-514583	WB,1:500
Santa Cruz Biotechnology	Mouse anti-RHOA	sc-418	WB,1:500
Santa Cruz Biotechnology	Mouse anti-USP4	sc-376000	WB,1:500
Santa Cruz Biotechnology	Mouse anti-USP8	sc-376130	WB,1:500
Santa Cruz Biotechnology	Mouse anti-USP11	sc-365528	WB,1:500
Santa Cruz Biotechnology	Mouse anti-USP25	sc-398414	WB,1:500
Santa Cruz Biotechnology	Mouse anti-Vimentin	sc-6260	WB,1:500
Santa Cruz Biotechnology	Mouse anti-ZEB2	sc-271984	WB,1:500
Proteintech	Rabbit anti-ARHGDIA	10509-1-Ig	WB,1:1000, IHC,1:100
Proteintech	Mouse anti-GAPDH	60004-1-Ig	WB,1:20000
Proteintech	Rabbit anti-ELAVL1	11910-1-AP	WB,1:1000
Proteintech	Rabbit anti-ALKBH5	16837-1-AP	WB,1:1000 IHC,1:400
Proteintech	Mouse anti-USP4	66822-1-Ig	WB,1:1000 IHC, 1:400
Proteintech	Rabbit anti-USP28	17707-1-AP	WB,1:1000
Proteintech	Rabbit anti-YTHDF1	17479-1-AP	WB,1:1000 Co-IP, 1:50 RIP, 1:50
Proteintech	Rabbit anti-YTHDF2	24744-1-AP	WB,1:1000 Co-IP, 1:50 RIP, 1:50
Sigma	Rabbit anti-METTL14	HPA038002	WB,1:2000 IHC,1:800
Synaptic Systems	Rabbit anti-m^6A	202003	IF,1:200

Table S2. Primers used for real-time PCR.

Accession number	Target gene	Primer sequence (5'-3') Forward	Reverse	Size (bp)	Tm (°C)
XM_011536968.2	*METTL3*	CTATCTCCTGGCACTCGCAAGA	GCTTGAACCGTGCAACCACATC	130	58.5
NM_001185077.3	*ARHGDIA*	GGATGAGCACTCGGTCAACTA	GGCCTCCTTGTACTTTCGCAG	103	58.5
NM_001419.3	*ELAVL1*	TTGGGCGGATCATCAACTCG	TCAAACCGGATAAACGCAACC	79	60
NM_003363.4	*USP4*	TGCAGCCTCAGAAGAAGAAGAA	ATGGTGGTGAAGAGCTCGATG	67	58.5
NM_001256799.3	*GAPDH*	AGAAGGCTGGGGCTCATTTG	AGGGGCCATCCACAGTCTTC	258	60

Table S3. Primers used for m^{6}A MeRIP-qRT-PCR analyses.

Gene	Sites	Primer sequence (5'-3') Forward	Reverse	Size (bp)	Tm (°C)
USP4	Site 1	TGGCCCAATAGACAACTCTGG	GCCTTCTACACAGCCGTACCA	142	60
USP4	Site 2	GGTGGATCTGGCTTTTCTGCT	CTCAAACACTGCAAAGCGGAG	128	60
USP4	Site 3	TTGGAAACCTGGGAAACACCT	CCCAGAGGGTTGTCTCTGTTGA	203	58.5
USP4	Site 4	AACTCTCGATCTACACTGGCCA	TCGTAGGCCTCAGATTCTTGC	83	58.5
USP4	Site 5	TGCAGCCTCAGAAGAAGAAGAA	ATGGTGGTGAAGAGCTCGATG	67	58.5
USP4	Site 6	CATTGCCGTGTCCAATCATTAT	TATCTGATCCTCAGAGGCCAGG	130	58.5
USP4	Site 7	ACCCAGCAGTTCTGTTATATCCC	AACAACTAGACGGGACACAGCA	155	58.5

Table S4. Primers used for RIP analyses.

Gene	Primer sequence (5'-3') Forward	Reverse	Size (bp)	Tm (°C)
USP4 pre-mRNA	ACTGAATGGTCTTTCCCCCTCT	TGCTTGGTCGTGTCCCTCC	138	60
USP4	CATTGCCGTGTCCAATCATTAT	TATCTGATCCTCAGAGGCCAGG	130	58.5

Table S5. Characteristics of human Prostate cancer patients.

Medical record no.	Age	Gleason score	WHO/ISUP classification	Lymphnode matastasis
2005653	71	9	5	-
2003086	60	9	5	-
2002795	52	8	4	-
1943107	61	8	4	-
1942249	76	8	4	-
1941411	53	9	5	-
1939213	67	9	5	-
1937473	76	9	5	-
1937115	62	9	5	-
1936722	65	8	4	-
1935640	63	6	1	-
1934978	72	9	5	-
1934972	62	9	5	-
1933891	74	8	4	-
1933609	66	7	3	-
1933570	69	7	3	-
1932399	68	9	5	-
1932055	63	8	4	-
1930183	57	9	5	-
1929314	81	7	3	-
1929013	53	7	3	-
1928395	55	8	4	-
1927577	62	7	3	-
1924448	70	6	1	-
1923382	66	6	1	-

Table S6. Primers used for methylation-specific PCR.

Gene	Sites	Primer sequence (5’-3’) Forward	Reverse	Size (bp)	Tm (°C)
USP4	Island 1	GATTTTAAAGTTTTTTTAGTGTCGG	CGAACATACTTCTTTATATTAACGAA	152	56.5
USP4	Island 2	TAATATTAGTTATTCGGGAGGTTGC	TTTAAAACGAAATTTCACTCTATCG	125	56.5
USP4	Island 3	GTTAGGGCGATAGAGTGAAATTTC	CATATTACTTATAAACGCGCATACG	169	55

Table S7. Expression levels of *42* USP members based on the TCGA dataset

	Expression levels			*p* value	
Genes	Normal	N0	N1	Normal-vs-N0	Normal-vs-N1
USP1	19.097	17.083	17.936	3.758200E-02	3.829400E-01
USP2	1.545	0.667	0.612	7.971200E-04	2.48399999999149E-05
USP3	17.678	19.599	21.357	5.400900E-03	1.719780E-03
USP4	25.347	21.511	22.844	3.972600E-04	1.262170E-02
USP5	59.691	53.198	52.657	5.820300E-02	3.853800E-02
USP6	0.294	0.333	0.425	6.517400E-01	2.235700E-02
USP7	42.897	47.399	53.116	6.797100E-03	1.93029999995264E-06
USP8	17.43	14.129	13.398	9.643400E-03	1.306350E-02
USP10	45.785	43.683	41.719	7.051800E-01	5.812800E-01
USP11	50.56	32.608	35.008	3.77200048973236E-10	3.28989999687224E-08
USP12	8.442	5.844	6.215	1.570430E-04	1.197230E-03
USP13	4.366	5.053	5.733	3.719400E-01	1.212770E-01
USP14	28.747	27.471	26.107	3.104200E-01	4.406400E-01
USP15	13.363	11.535	13.129	7.735500E-03	4.865400E-01
USP16	33.515	32.095	31.404	9.122100E-02	2.533800E-01
USP18	8.814	10.098	10.285	3.81470000000439E-05	2.44099999999747E-05
USP19	31.391	32.095	32.774	8.967600E-01	8.175600E-01
USP20	28.049	27.033	28.854	3.711700E-02	4.020700E-04
USP21	19.925	20.868	21.133	9.778400E-01	6.420200E-02
USP22	69.829	89.058	93.404	4.85649999992788E-07	4.42329999994495E-06
USP24	9.078	9.429	9.614	3.534400E-01	5.075000E-01
USP25	14.543	11.502	11.586	1.5198899999902E-05	2.168100E-04
USP28	13.847	10.396	12.239	3.71829999999962E-05	3.127400E-01
USP30	11.186	10.145	10.438	8.824600E-03	5.881800E-01
USP31	5.058	3.992	4.478	2.174000E-02	6.630800E-01
USP32	9.208	8.375	9.763	2.836000E-01	2.592400E-01
USP33	38.25	41.1	39.874	1.184060E-02	1.728360E-01
USP34	56.891	52.26	54.557	1.437380E-01	8.857200E-01
USP35	2.603	2.355	2.792	8.641600E-02	1.926570E-01
USP36	21.229	22.993	25.261	1.841380E-01	9.188400E-04
USP37	4.208	3.388	3.561	1.132420E-02	2.622000E-01
USP38	11.026	10.621	10.559	4.891200E-01	8.117000E-01
USP39	39.83	35.638	38.784	5.097700E-02	1.979630E-01
USP40	34.711	43.115	41.726	4.021000E-03	3.033500E-02
USP42	4.549	5.624	6.435	1.664710E-03	5.69649999948574E-07
USP43	8.735	9.116	9.296	5.241800E-01	3.114600E-01
USP44	0.399	0.161	0.116	2.79230000010955E-07	2.1378000003569E-07
USP45	5.468	5.651	5.581	6.535800E-02	2.239800E-02
USP46	6.001	4.872	4.636	1.365610E-02	2.320200E-02
USP47	31.498	28.808	28.114	1.756120E-02	8.458600E-01

USP48	23.304	22.778	24.227	8.427800E-01	2.741000E-01
USP49	0.327	0.354	0.375	1.801020E-01	8.242400E-02

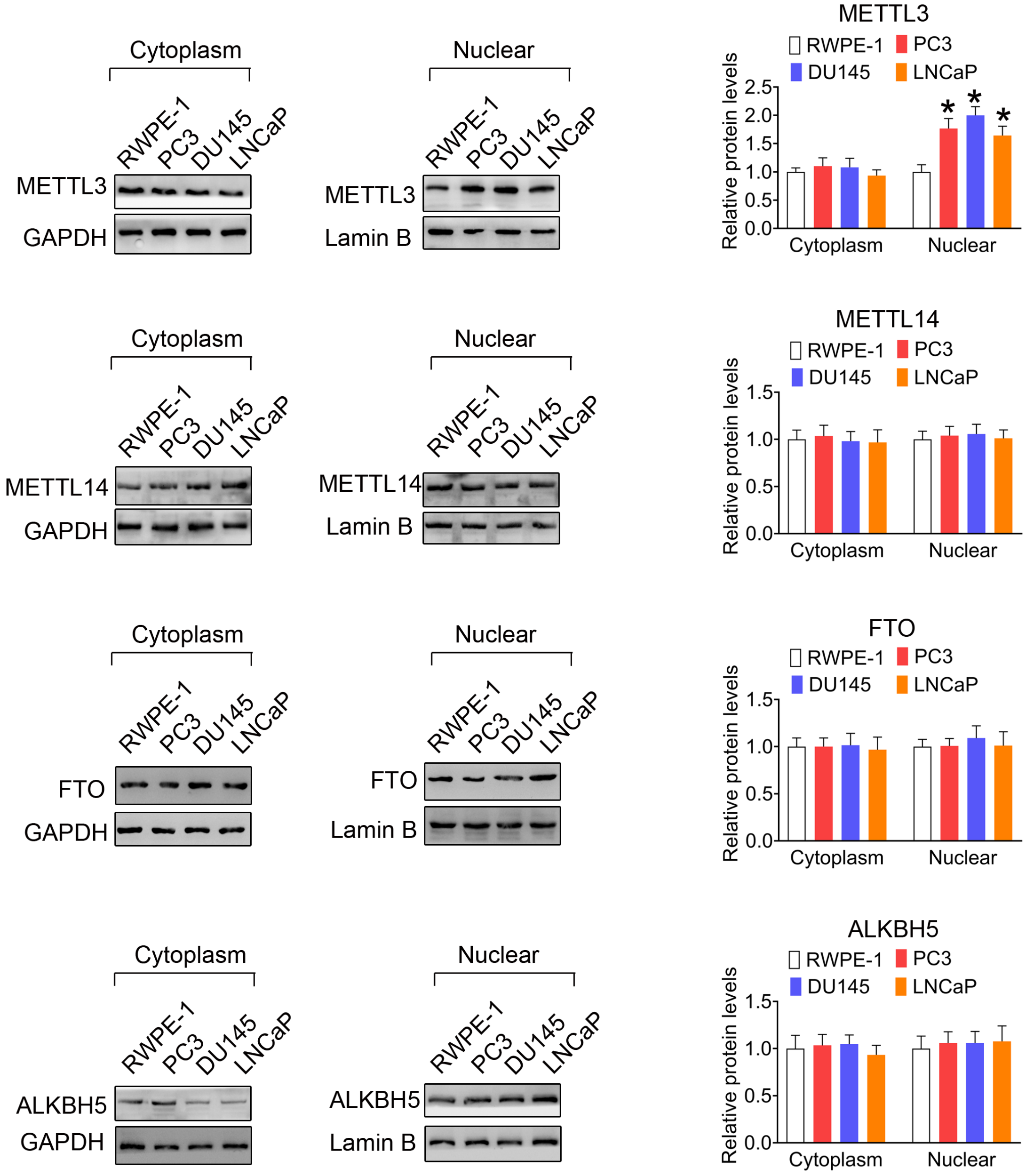

Figure S1. METTL3 but not METTL14, FTO, and ALKBH5 was markedly upregulated in the nuclei of prostate cacer (PCa) cells relative to RWPE-1 cells. Distribution of METTL3, METTL14, FTO, and ALKBH5 in cytoplasm and nuclear were examined by western blotting and quantitatively analyzed. Data were presented as means ± SEM (n = 3) where relevant, **p* < 0.05 vs. the RWPE-1 cells.

.

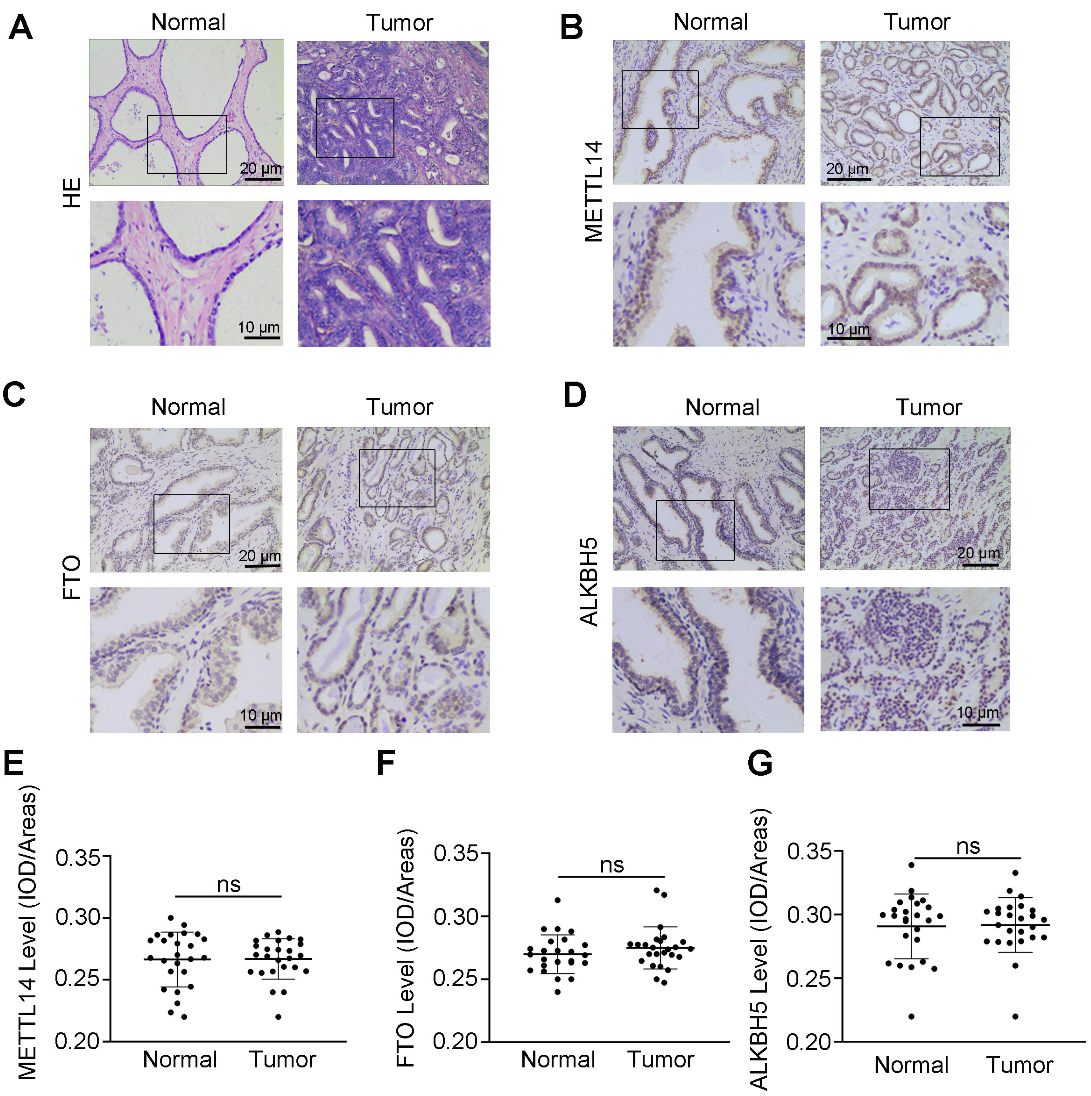

Figure S2. No significant difference of METTL14, FTO, or ALKBH5 expression is observed in human prostate cancer (PCa) tissues. **A,** Representative H&E staining results of human PCa tissue were shown. **B-D,** Immumohistochemical staining was performed to evaluate the expression of METTL14 (**B**), FTO (**C**), and ALKBH5 (**D**) in 25 paired human PCa tissues and their adjacent normal prostate tissues. **E-G,** The expression levels of target protein were analyzed by calculating the integrated optical density per area (IOD/area). Data were presented as means ± SEM (n = 25). An "ns" denotes no statistically significant difference between the testing groups.

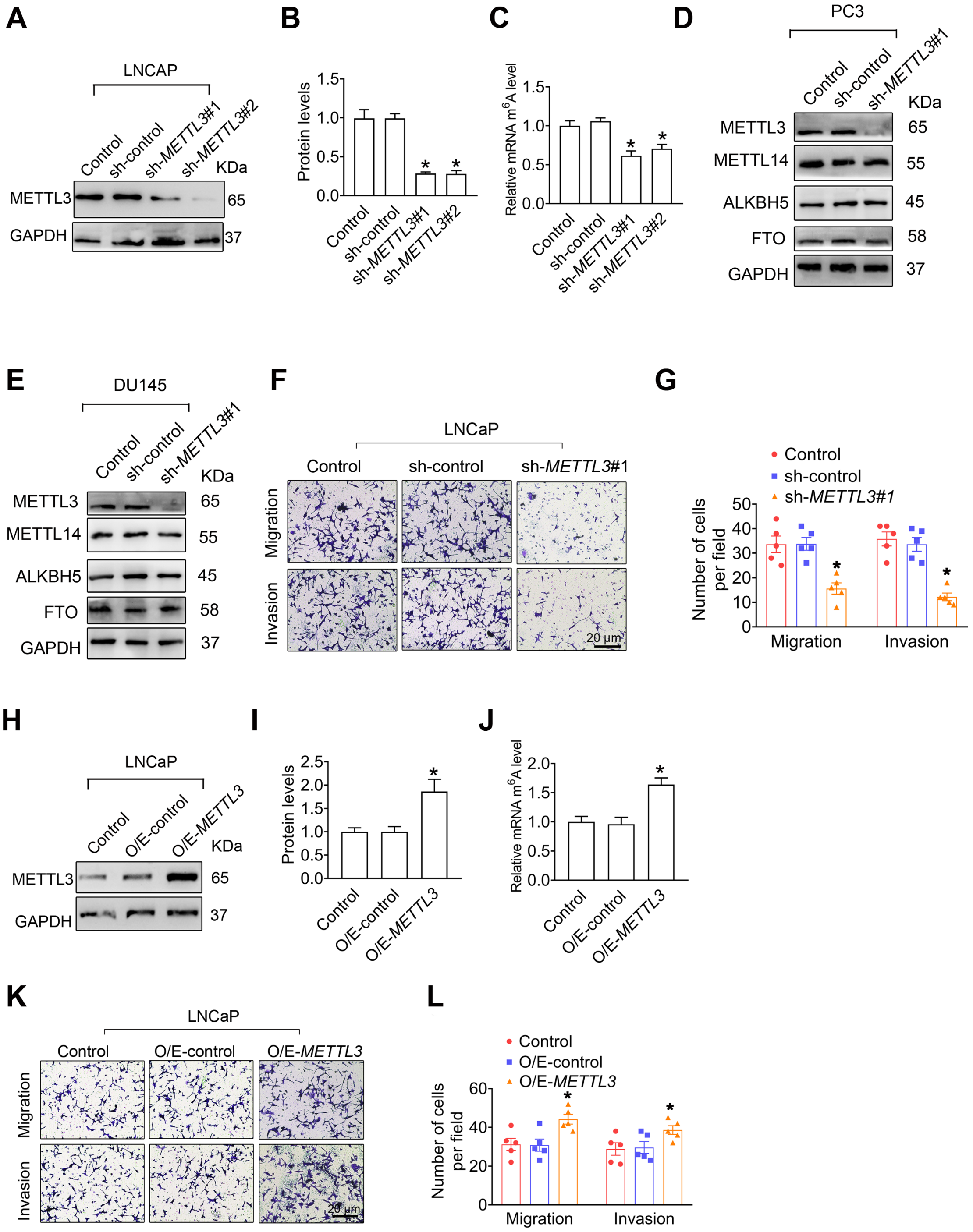

Figure S3. METTL3 promotes prostate cancer (PCa) metastasis. A-C, Two independent shRNA targeting *METTL3* (sh-*METTL3*#1 and sh-*METTL3*#2) were separately transfected into LNCaP cells. Protein levels of METTL3 were examined by western blotting and quantitatively analyzed (**A, B**), and cellular m^6A levels were measured by ELISA-based m^6A quantitative analyses (**C**). **D-E,** PC3 and DU145 cells were transfected with sh-*METTL3*#1, respectively. Western blotting was used to examine protein expression of other m^6A-modification related proteins. **F-G,** The migration and invasion abilities of indicated LNCaP cells were evaluated. **H-L,** Overexpression constructs of *METTL3* (O/E-*METTL3*) were stably transfected into

LNCaP cells. Protein levels of METTL3 were examined by western blotting and quantitatively analyzed (**H, I**), and cellular m^6A levels were measured by ELISA-based m^6A quantitative analyses (**J**). The migration and invasion abilities of indicated cells were assessed (**K, L**). Data were presented as means ± SEM where relevant, *p < 0.05 vs. the control cells.

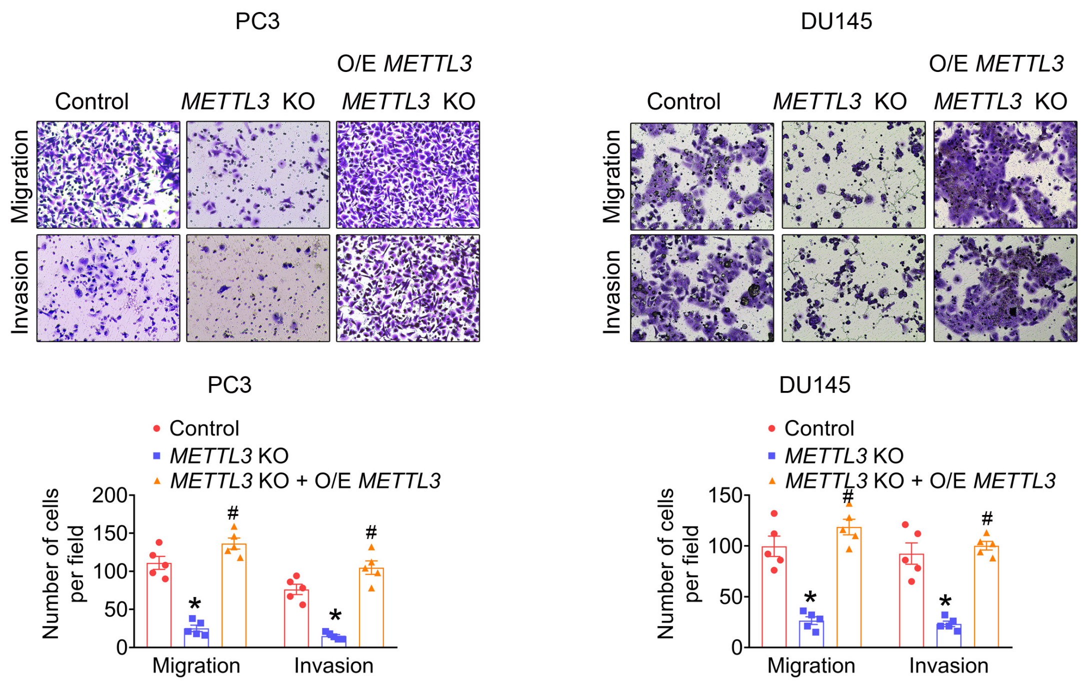

Figure S4. METTL3 is required for migration and invasion of PCa cells. METTL3 knockout (KO) in PCa cells was accomplished by using the lentiviral-based CRISPR gene editing system (pLentiCRISPR v2), and then these cells were transfected with METTL3 expression plasmid. The migration and invasion abilities of indicated cells were evaluated. Data were presented as means ± SEM (n=5), *$p < 0.05$ vs. the control cells, # $p < 0.05$ vs. the *METTL3* KO cells.

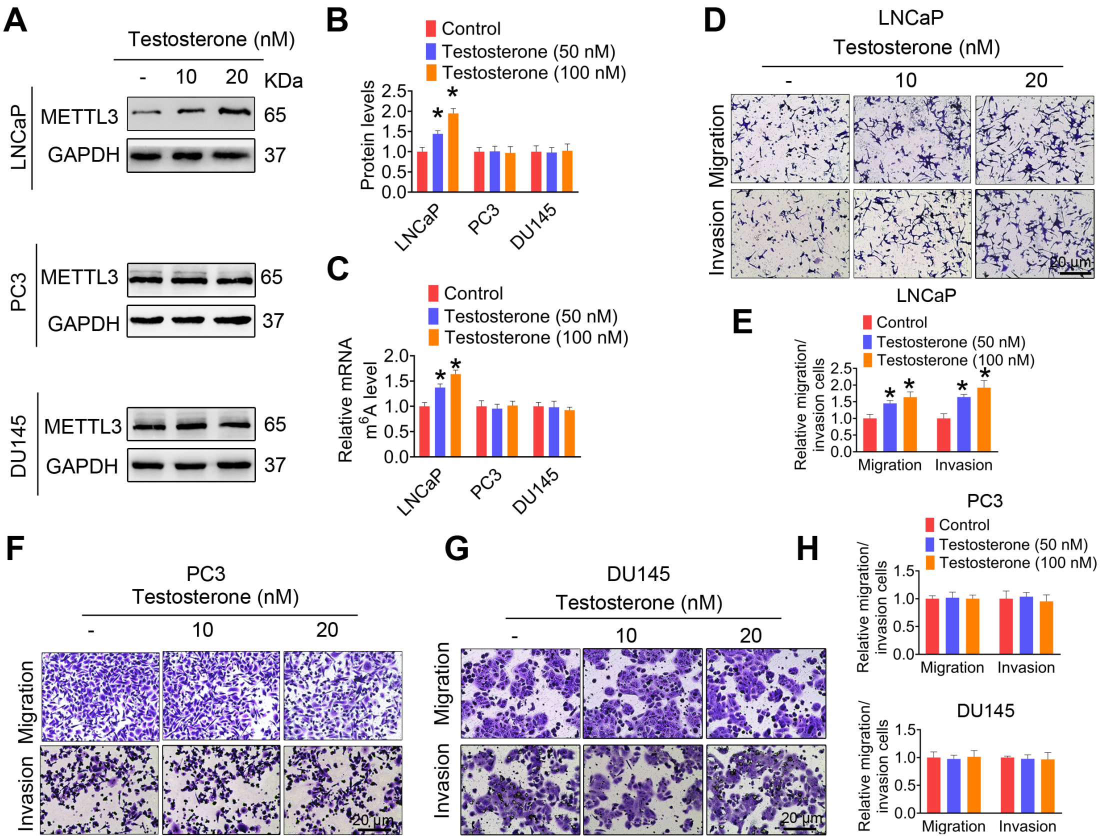

Figure S5. Testosterone promotes expression of METTL3 in androgen-sensitive LNCaP cells. A-C, PC3, DU145, and LNCaP cells were treated with testosterone for 24 h, respectively. The cell lysates were subjected to western blotting to detect the expression of METTL3 (**A, B**), and cellular m^{6}A levels were measured by ELISA-based m^{6}A quantitative analyses (**C**). **D-H,** The migration and invasion abilities of indicated cells were evaluated. Representative images (**D-G**) and quantification (**E, H**) of the cell migration and invasion assay results were shown. Data were presented as means ± SEM (n = 3) where relevant, *$p < 0.05$ vs. the control cells.

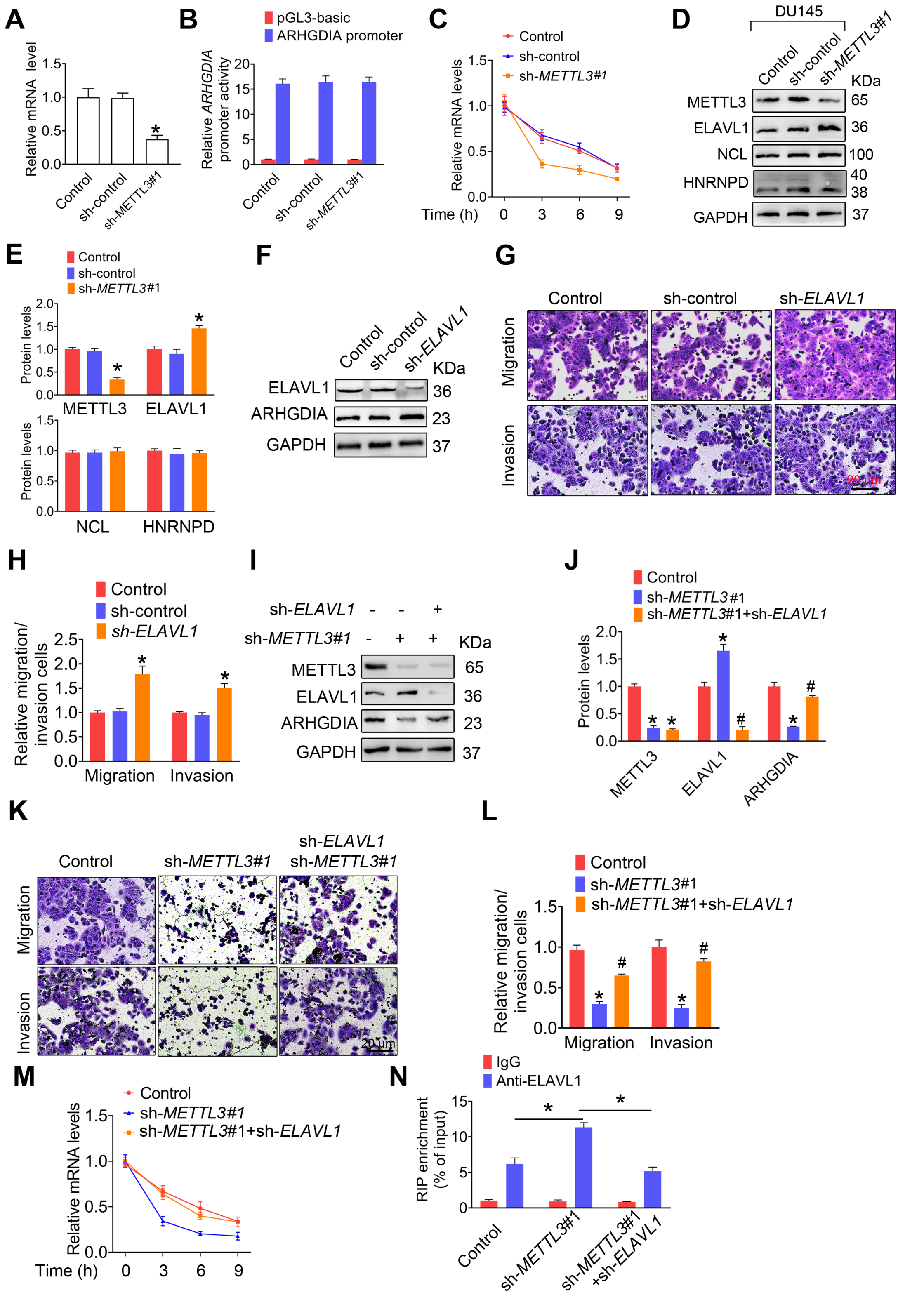

Figure S6. ELAVL1 reduces *ARHGDIA* mRNA stability and prostate cancer (PCa) metastasis. A-E, DU145 cells were transfected with shRNA targeting *METTL3* (sh-*METTL3*#1). The *ARHGDIA* mRNA levels in indicated cells were determined by qRT-PCR assay (**A**). The *ARHGDIA* promoter constructs were transfected into PC3 cells, and luciferase activity was measured (**B**). DU145 cells were treated with actinomycin D (5 μg/mL) for 2 h, followed by measurement of *ARHGDIA* mRNA

levels at indicated times (**C**). Protein levels of ELAVL1, NCL, and HNRNPD were examined by western blotting and quantitatively analyzed (**D, E**). Data were presented as means ± SEM (n = 3), * $p < 0.05$ vs. the control cells. **F**-**H,** DU145 cells were transfected with sh-*ELAVL1*. Protein levels of ELAVL1 and ARHGDIA were examined by western blotting (**F**). The migration and invasion abilities of indicated cells were evaluated. Representative images (**G**) and quantification (**H**) of the cell migration and invasion assay results were shown. Data were presented as means ± SEM (n = 5), * $p < 0.05$ vs. the control cells. **I**-**M,** DU145 cells were transfected with sh-*METTL3* before transfection with sh-*ELAVL1*. Protein levels of METTL3, ELAVL1, and ARHGDIA were examined by western blotting and quantitatively analyzed (**I, J**). The migration and invasion abilities of indicated cells were evaluated. Representative images (**K**) and quantification (**L**) of the cell migration and invasion assay results were shown. DU145 cells were treated with actinomycin D (5 μg/mL) for 2 h, followed by measurement of *ARHGDIA* mRNA levels at indicated times (**M**). Data were presented as means ± SEM (n = 3), * $p < 0.05$ vs. the control cells, # $p < 0.05$ vs. the sh-*METTL3*-treated cells. **N,** ELAVL1 was immunoprecipitated, followed by qRT-PCR assay to evaluate the association of the *ARHGDIA* transcripts with ELAVL1 protein. Data were presented as means ± SEM (n = 3), * $p < 0.05$.

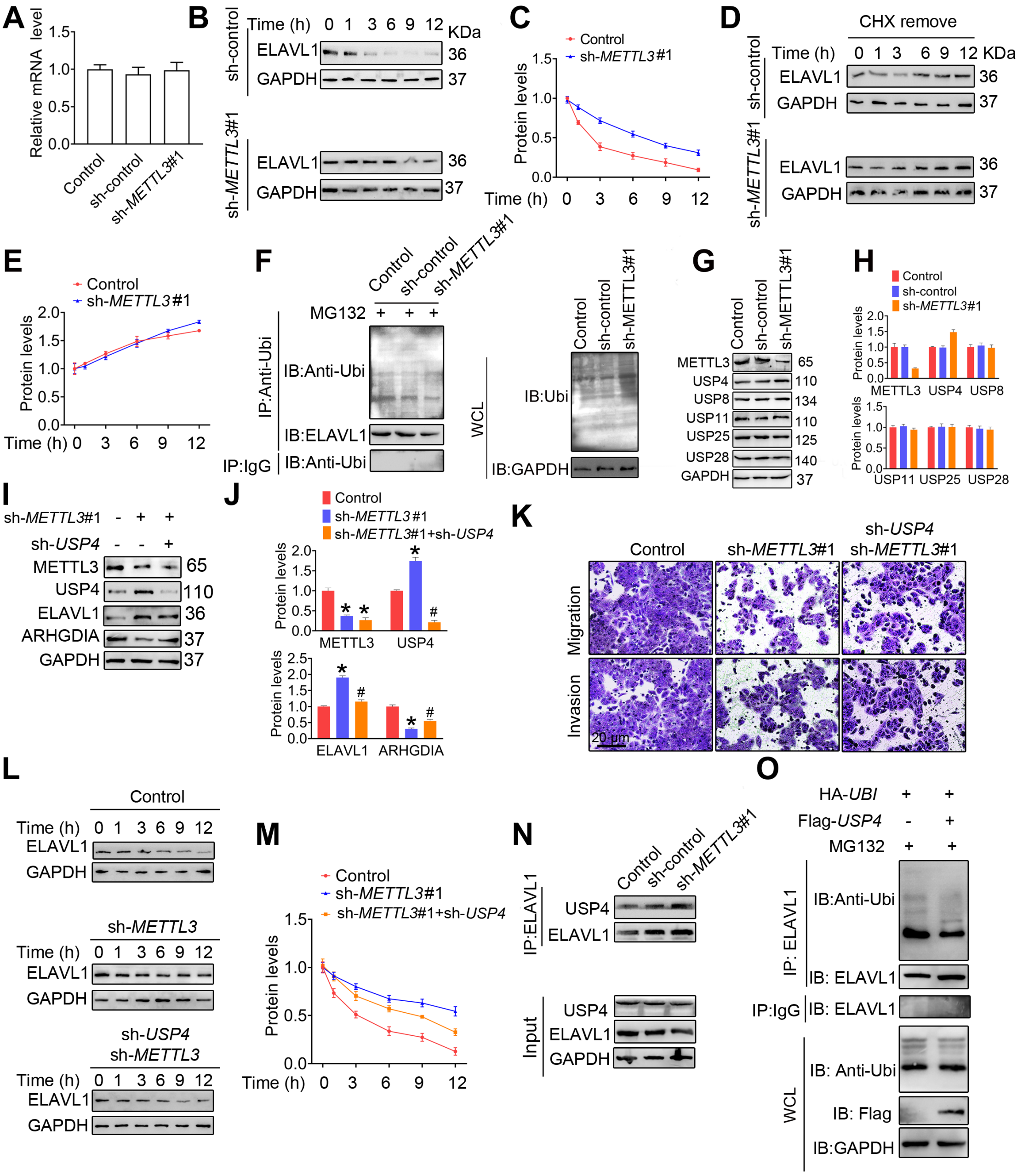

Figure S7. USP4 is an METTL3 downstream effector and mediates ELAVL1 protein stability. DU145 cells were transfected with shRNA targeting *METTL3* (sh-*METTL3*#1). **A,** The *ELAVL1* mRNA levels in indicated cells were determined by qRT-PCR assay. **B-C,** The indicated DU145 cells were pretreated with cycloheximide (CHX, 10 μg/mL) for 3 h, followed by measurement of ELAVL1 protein levels at indicated times. **D-E,** DU145 cells were treated with CHX for 12 h. After washing out cycloheximide, cells were cultured for the indicated times. ELAVL1 synthesis levels were detected by western blotting and quantitatively analyzed. **F,** DU145 cells were treated with MG132 for 6 h. Lysates from the indicated cells were subjected to coimmunoprecipitation (Co-IP) assay with anti-ELAVL1 antibody, and the blots were then probed with anti-ubiquitin (UB) antibody for detection of ubiquitination of ELAVL1. **G-H,** Protein levels of USP4, USP8, USP11, USP25, and USP28 in indicated cells were examined by western blotting and quantitatively analyzed. Data

were presented as means ± SEM (n = 3), * $p < 0.001$ vs. the control cells. **I**-**N,** DU145 cells were transfected with sh-*METTL3* before transfection with sh-*ELAVL1*. Protein levels of METTL3, USP4, ELAVL1, and ARHGDIA were examined by western blotting and quantitatively analyzed (**I, J**). The migration and invasion abilities of indicated cells were evaluated (**K**). The indicated cells were pretreated with cycloheximide (CHX, 10 μg/mL) for 3 h, followed by measurement of ELAVL1 protein levels at indicated times (**L, M**). Lysates from the indicated cells were subjected to Co-IP with anti-ELAVL1, and the blots were probed with anti-USP4 antibody (**N**). **O,** DU145 cells were transfected with indicated plasmids, and ubiquitination of ELAVL1 was detected by Co-IP assay. Data were presented as means ± SEM (n = 3), * $p < 0.05$ vs. the control cells, # $p < 0.05$ vs. the sh-*METTL3*-treated cells. WCL: whole cell lystate.

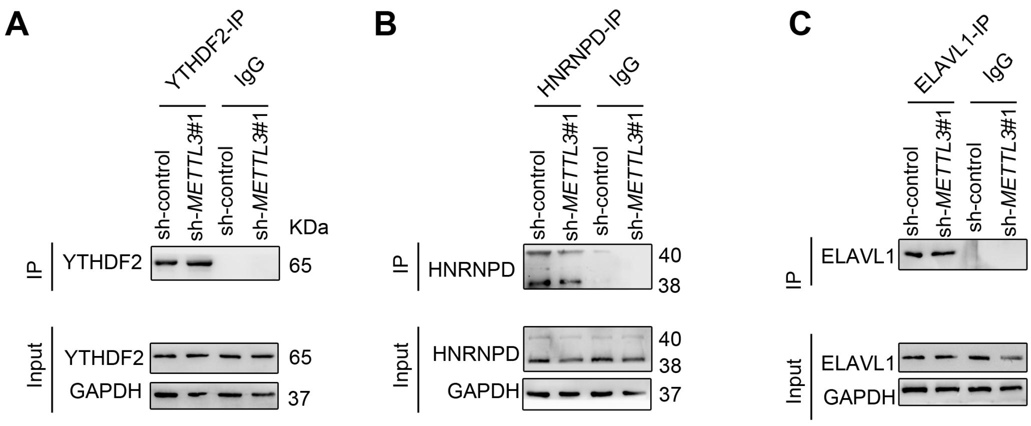

Figure S8. Immunoprecipitated YTHDF2/HNRNPD/ELAVL1 protein levels are examined by western blotting. PC3 cells were transfected with shRNA targeting *METTL3* (sh-*METTL3*#1). **A-C,** Lysates from the PC3 cells were subjected to immunoprecipitation with anti-YTHDF2/HNRNPD/ELAVL1, and the association of the *USP4* transcript with each protein was determined by qRT-PCR. Immunoprecipitated YTHDF2 (**A**), HNRNPD (**B**), or ELAVL1 (**C**) were examined by western blotting.

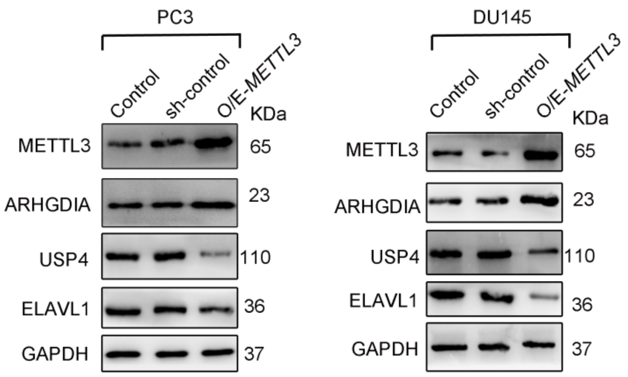

Figure S9. METTL3-USP4-ELAVL1-ARHGDIA regulatory axis mediates prostate cancer (PCa) metastasis. Overexpression constructs of *METTL3* (O/E-*METTL3*) were stably transfected into PC3 and DU145 cells, respectively. Protein levels of METTL3, USP4, ELAVL1, and ARHGDIA were determined by western blotting.

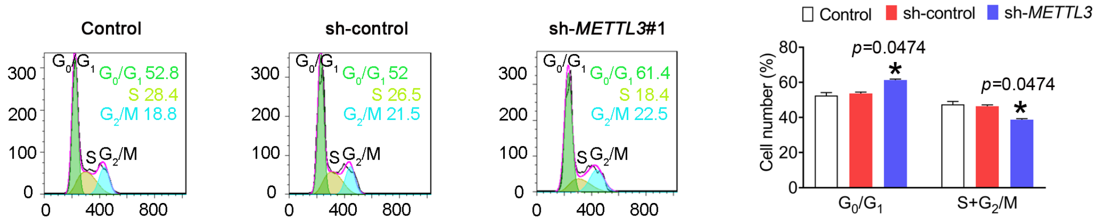

Figure S10. Knockdown of METTL3 exerted inhibitory effects on cell proliferation of PC3 cells. PC3 were transfected with shRNA targeting *METTL3* (sh-*METTL3*#1), respectively; cells were stained with PI and then analyzed by flow cytometry. Data were presented as means ± SEM (n = 3), * $p < 0.05$ vs. the control cells.

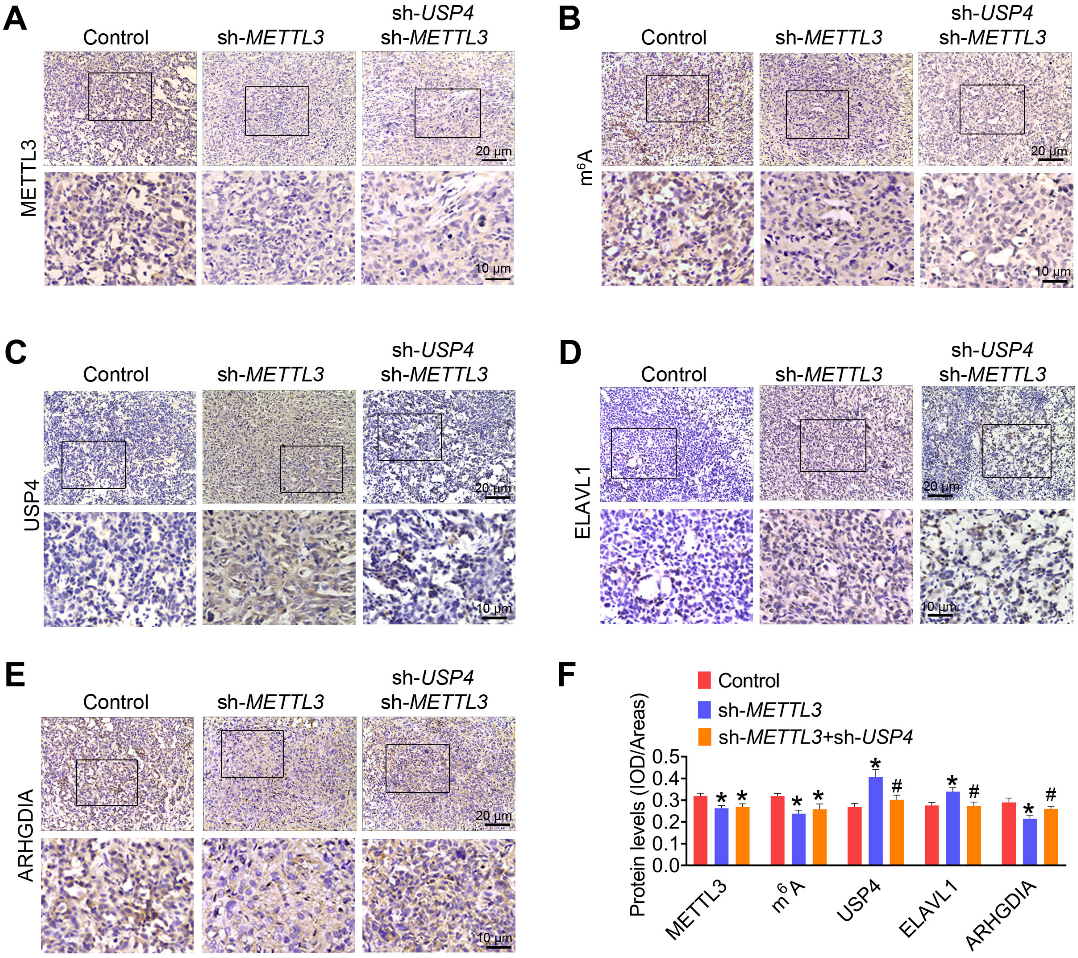

Figure S11. m^{6}A methylation regulates in vivo cancer progression. PC3 cells were transfected with shRNA targeting *METTL3* (sh-*METTL3*) before transfection with sh-*USP4*. Athymic nude mice were subcutaneously injected into the right axillary region of each mouse with indicated cells. **A-F,** Eight weeks after cell injection, the xenograft tumor tissues obtained from athymic nude mice were subjected to immumohistochemical staining assay for evaluating METTL3 (**A**), m^{6}A modification (**B**), USP4 (**C**), ELAVL1 (**D**), and ARHGDIA (**E**) levels; the m^{6}A levels and the expression levels of the target proteins were analyzed by calculating the integrated optical density per area (**F**). Data were presented as means ± SEM (n = 6), * $p < 0.05$ vs. the control cells, # $p < 0.05$ vs. the sh-*METTL3*-treated cells.
